# Acquisition of daptomycin resistance in patients results in decreased virulence in *Drosophila*

**DOI:** 10.1128/iai.00594-24

**Published:** 2025-05-23

**Authors:** Brigitte Lamy, Frédéric Laurent, Carolina J. Simoes Da Silva, Ashima Wadhawan, Elizabeth V. K. Ledger, Camille Kolenda, Patricia Martins Simoes, Andrew M. Edwards, Marc S. Dionne

**Affiliations:** 1 Hôpitaux Universitaires Paris Seine Saint-Denis, APHP, Université Sorbonne Paris Nord 27097, Villetaneuse, France; 2 Infection Antimicrobials Modelling Evolution (IAME), INSERM, Paris, France; 3 Centre for Bacterial Resistance Biology, Imperial College London 4615, London, United Kingdom; 4 Centre International de Recherche en Infectiologie, INSERM, Université Claude Bernard Lyon 1, CNRS, École Normale Supérieure de Lyon 236341, Lyon, France; 5 Laboratoire de Bactériologie, Institut des Agents Infectieux, Centre National de Référence des Staphylocoques, Hôpital de la Croix-Rousse, Hospices Civils de Lyon 379398, Lyon, France; 6 Department of Life Sciences, Imperial College London 98455, London, United Kingdom; Universite de Geneve, Geneve, Switzerland

**Keywords:** *Staphylococcus aureus*, *Drosophila melanogaster*, host-pathogen interaction, virulence, antimicrobial resistance, link between antimicrobial resistance and virulence, prophenoloxidase, antimicrobial peptides, daptomycin

## Abstract

*Staphylococcus aureus* can acquire antimicrobial resistance, which in turn may affect its pathogenic potential. Using a panel of paired clinical isolates collected before and after daptomycin resistance acquisition, most frequently through a single *mprF* mutation, we show a relationship between increasing daptomycin minimum inhibitory concentration and reduced virulence in a *Drosophila* systemic infection model. Analyzing toxin production, *in vitro* bacterial growth characteristics, and cell surface properties, we failed to link daptomycin resistance-related attenuated virulence to either reduced virulence factor production, reduced fitness, or any of the cell surface characteristics investigated. Competition assays in *Drosophila* also did not support any altered ability in immune evasion. Instead, using a panel of mutant flies defective for various immune components, we show that this daptomycin resistance-related attenuated virulence is mostly explained by greater susceptibility to the activity of *Drosophila* prophenoloxidase, a tyrosinase involved in melanization, but not to antimicrobial peptides or Bomanin antimicrobial effectors. Further investigation could not link daptomycin resistance-related attenuation of virulence to differential susceptibility to reactive oxygen species or quinones prominently associated with phenoloxidase bacterial-killing activity. Taken together, it appears that daptomycin resistance attenuates *Staphylococcus aureus* virulence through enhanced sensitivity to phenoloxidase based on a complex mechanism. Our study provides new insights into the understanding of the crosstalk between antimicrobial resistance, escape from immune killing, and virulence.

**IMPORTANCE:**

This study advances current knowledge in the field of host-microbe interactions and antimicrobial resistance by exploring crosstalk between antimicrobial resistance and virulence. It shows how acquiring antimicrobial resistance can alter bacterial virulence and helps shape virulence. Relative to the parental staphylococcal strain, daptomycin-resistant clinical isolates most often varied by one single mutation in a gene involved in the composition of the bacterial membrane, and these strains were much less virulent when fruit flies were infected. The difference in virulence is unrelated to changes in bacterial toxin production, bacterial growth, immune evasion, or cell surface properties. Instead, resistant strains were more vulnerable to a host proenzyme involved in the antibacterial melanization response, an important response deployed throughout the arthropods. We predict that daptomycin resistance forces staphylococci to alter the composition of their cell surface, which causes the bacteria to become more vulnerable to killing by melanization.

## INTRODUCTION

*Staphylococcus aureus* is a major cause of bacteremia. It has an estimated incidence of 9.3–65 cases/100,000 per year and a 30-day mortality rate between 18% and 29% (1, 2). Some sources of staphylococcal bacteremia stand out as difficult-to-treat infections (e.g., infective endocarditis or osteomyelitis), and the choice of treatment primarily relies on antimicrobial susceptibility (3–5). While bacteremia caused by methicillin-sensitive *S. aureus* (MSSA) is usually treated with antistaphylococcal penicillins, those caused by methicillin-resistant *S. aureus* (MRSA) or MSSA in penicillin-allergic patients (5, 6) are treated with daptomycin, vancomycin, or linezolid (4, 7, 8). However, treatment with both vancomycin and daptomycin can be complicated by resistance that can arise via spontaneous mutations that occur during treatment (9, 10).

Acquisition of antimicrobial resistance appears to be frequently associated with changes in virulence (11–13). For example, vancomycin-intermediate *Staphylococcus aureus* can exhibit reduced fitness as a result of a thickened cell wall, enhanced capsular polysaccharide production, and dysfunction of the Agr virulence regulatory system (14–21). The consequences of daptomycin resistance on fitness and virulence are not well established.

Daptomycin compromises bacterial surface integrity by targeting cell wall biosynthesis and membrane disruption, which may drive bacteria to alter cell surface characteristics and consequently affect immune host defense recognition (22, 23). Moreover, daptomycin shares a similar mechanism of action to host innate immune cationic antimicrobial peptides (AMPs), which may facilitate cross-resistance between daptomycin and these important immune effectors (24–26). Cross-resistance to host cationic AMPs, e.g., cathelicidin LL-37 or hNP-1, has been reported in some clinically derived daptomycin-resistant *S. aureus* isolates in *in vitro* studies (24–26). However, daptomycin resistance has been reported to result in attenuated virulence in a zebrafish model (e.g., reference 26).

*Drosophila melanogaster* is a well-established model for the study of antibacterial immunity (27). *Staphylococcus aureus* peptidoglycan is detected via the circulating pattern recognition receptor PGRP-SA, resulting in the activation of the Toll signaling pathway and an antibacterial melanization response (28). The Toll pathway drives the production of antimicrobial peptides, while the melanization response depends on phenoloxidase activity. Of these responses, melanization appears to be the most important mechanism of defense against *Staphylococcus aureus* infection (29). This response depends on the conversion of prophenoloxidase to active phenoloxidase by serine protease cleavage. Phenoloxidase then converts tyrosine to dopaquinone, which polymerizes to form melanin. It is unclear whether dopaquinone, associated reactive oxygen species, or melanin itself is the relevant killing mechanism (30, 31).

It is commonly acknowledged that some host immune effectors and antimicrobials share common mechanisms of action, suggesting potential mechanistic links between virulence and antimicrobial resistance (32, 33). Although there is growing evidence of a trade-off between daptomycin resistance and pathogenic potential, the associated mechanisms are still poorly understood. This is further confounded by the fact that investigation is often limited to single clinical paired strains or laboratory-generated daptomycin-resistant strains (21, 26, 34, 35). Thus, the ability to generalize previous findings is limited by the use of one or very few strain pairs. Moreover, many studies focus on the correlation between AMR and *in vitro* proxies for virulence, such as gene expression, but the impact of these changes on *in vivo* virulence is often unexplored. Finally, the molecular determinants underlying the interaction between antimicrobial resistance and pathogenic potential are often unclear. Understanding these determinants is informative regarding the fundamental biology of treatment and pathogenesis and may help identify new antimicrobial strategies to either restore antimicrobial sensitivity or reduce virulence.

Here, we were interested in exploring the trade-off between daptomycin resistance and staphylococcal virulence as a strategy to deepen our understanding of staphylococcal pathogenicity and inform efforts to devise new antimicrobial strategies. Using a panel of *S. aureus* clinical strains recovered from bacteremia before and after daptomycin resistance acquisition, we investigated the impact of daptomycin resistance on virulence using a *Drosophila* systemic infection model to decipher crosstalk between daptomycin resistance and staphylococcal pathogenesis. We also investigated the impact of daptomycin resistance on the interaction between *S. aureus* and innate immunity. This led to the discovery that daptomycin resistance is associated with increased susceptibility to phenoloxidase-mediated killing.

## RESULTS

### The acquisition of daptomycin resistance during treatment results in reduced virulence

To understand the consequences of *in vivo* acquired daptomycin resistance on staphylococcal virulence, we collected 11 pairs of isolates from patients with *S. aureus* endocarditis in whom daptomycin resistance arose during treatment. Isolates collected before treatment were all susceptible to daptomycin, and those collected after were resistant to the lipopeptide antibiotic.

In keeping with the literature, daptomycin resistance was associated with two- to eightfold increases in daptomycin MIC (from 0.125 to 0.5 to 1–2 mg/L) and oxacillin MIC decreases in 9/11 pairs (2- to 64-fold reduction), while vancomycin MICs were unchanged (Table 1) (36–39). Whole genome sequencing found 7/11 pairs in which susceptible and resistant strains differed only by single mutations in the multipeptide resistance factor (*mprF*) gene commonly associated with daptomycin resistance (40, 41). One pair (pair D) had acquired mutations in *mprF* and *rpoB*, while pair J had only acquired a mutation in *rpoB*. Pairs B and H had acquired multiple insertions, deletions, and single nucleotide polymorphisms in addition to *mprF* mutations (*n* = 3 and *n* = 13, respectively) (Table 1). Thus, the isolates assembled here are representative of previously reported clinical daptomycin-resistant strains (10, 42, 43). Furthermore, since many isolates differed by a single mutation, we were able to investigate the impact of daptomycin resistance on virulence in the absence of potentially confounding mutations.

**TABLE 1:** Characteristics of the clinical strains included in this study^*e*^^,^^*f*^

Pair	Strain	Daptomycin susceptibility	Multilocus sequence type^*a*^	Amino acid change in protein	MIC daptomycin (mg/L)^*d*^	MIC vancomycin (mg/L)^*d*^	MIC oxacillin (mg/L)^*d*^
A	ST20200244	S	5	–	0.125–0.25	2	2–
	ST20200225	R	5	MprF L826F	1–2	2	0.25–0.5
B	ST20171659	S	5	–	0.5	2	128
	ST20171658	R	5	MprF S295L three other differences^*b*^	2	2	4–16
C	ST20150287	S	582	–	0.5	2–4	≤0.25
	ST20150288	R	582	MprF L826F	2	2–4	≤0.25
D	ST20171642	S	8	–	0.25	2	16
	ST20171644	R	8	MprF S337L RpoB, H481R	2	2	≤0.25
E	ST20200611	S	7	–	0.5	2	0.5–1
	ST20200408	R	7	MprF T423R	1–2	2	0.25–0.125
F	ST20200655	S	8	–	0.125–0.25	2	32
	ST20200654	R	8	MprF P314L	1	2	8
G	ST20171810	S	5	–	0.25–0.5	2	16
	ST20171811	R	5	MprF L826F	2	2	8
H	ST20160260	S	8	–	0.25–0.5	2	128
	ST20160261	R	8	MprF, P314L 13 other differences^*c*^	1–2	4	32
I	ST20182004	S	398	–	0.25	2	≤0.25
	ST20181963	R	398	MprF, L826F	2	2–4	≤0.25
J	ST20141980	S	8	–	0.25	2	8–16
	ST20141981	R	8	RpoB, Q468L	1	2	64
K	ST20201745	S	5	–	0.25	2	32–64
	ST20201688	R	5	MprF, L826F	2	2	32–64

^*a*^ Multilocus sequence typing was performed using assemblies (https://github.com/tseemann/mlst) and PubMLST (https://pubmlst.org/).

^*b*^ Pair B: lipoate-protein ligase A (365delA, N122fs), glutamyl endopeptidase (907dupA, T303fs), and monovalent cation antiporter-3, MnhG (266dupG, M90fs).

^*c*^ Putative phosphopentomutase (1029dupTAT p.Y343dup), superoxide dismutase (207G > T p.M69I), immunoglobulin G binding protein A (97G > A p.A33T), putative ABC transporter permease (494T > C, L165P), hypothetical protein (c.634dupA, I212fs), hypothetical protein (25A > T, K92*), 6-carboxyhexanoate--CoA ligase (578C > T, P193L), checkpoint controller nucleotide-binding protein (392G > T, R131I), hypothetical protein (7G > A, D3N), MerR family transcriptional regulator (345_355delAATGAAGCGTC, M116fs), DNA-directed RNA polymerase subunit beta (1441C > T, H481Y), transcriptional regulator (115G > A, A39T), and transcriptional regulator (179T > C, L60S).

^*d*^ When replicates exhibited some variability in MIC values, results are provided as a range.

^*e*^ For the purpose of clarity, we named daptomycin-resistant strains with daptomycin MIC > 1 µg/mL even though the official term is daptomycin non-susceptible (44).

^*f*^ –, Not applicable.

**Fig 1:**
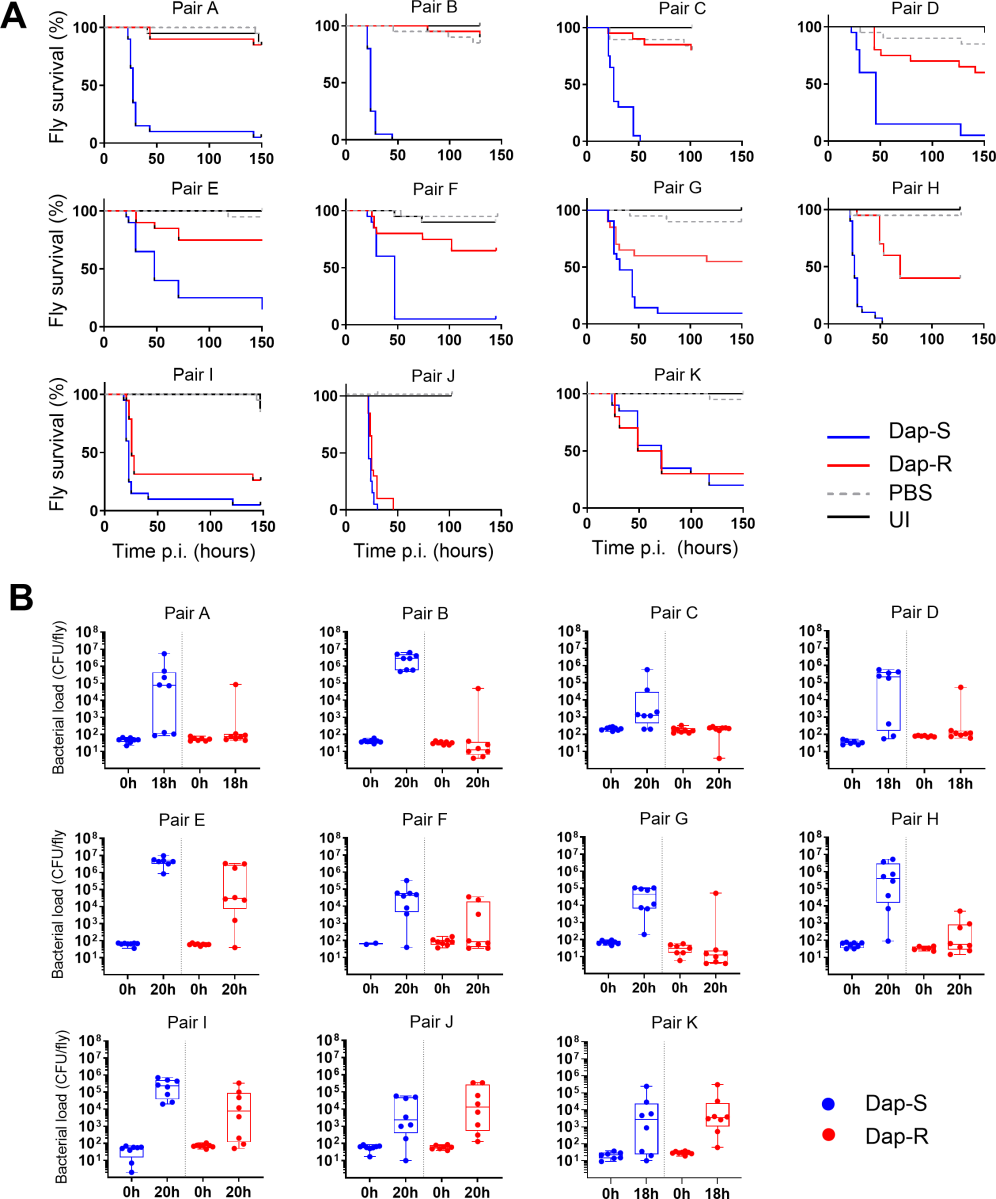
Daptomycin-resistant strains exhibit lower virulence and reduced growth in the fly relative to susceptible isolates. (**A**) Survival of wild-type (*w^1118^*) flies infected with Dap-S strain and Dap-R paired isolate. Each graph corresponds to one experiment with 20 flies per condition and is representative of at least two independent experiments with similar results. *P*-values were calculated using the log-rank test. *P*-values for survival differences with Dap-R isolate vs Dap-S paired isolate: pairs A–F and H, *P* ≤ 0.0001; pair G, *P* = 0.007; pair I, *P* = 0.0006; pair J, *P* = 0.046; pair K, *P* = 0.636; and PBS and UI, *P* > 0.05. (**B**) Strain colony counts at 0 h (input inoculum) and 18–20 h after injection of wild-type (*w^1118^*) flies with Dap-S strain and Dap-R paired isolate. Bacterial counts presented correspond to one experiment and are representative of two independent experiments. Boxplots represent the median, percentiles 25 and 75, and whiskers to the minimum and maximum values. *P*-values for bacterial numbers at 18–20 h post-infection (Dap-R vs Dap-S paired strain) were calculated using the Mann-Whitney test: pair A, *P* = 0.0104; pair B, *P* = 0.0002; pair C, *P* = 0.0342; pair D, *P* = 0.0779; pair E, *P* = 0.004; pair F, *P* = 0.0277; pair G, *P* = 0.0017; pair H, *P* = 0.0011; pair I, *P* = 0.0207; pair J, *P* = 0.36; and pair K, *P* = 0.64. Dap-S, daptomycin susceptible; Dap-R, daptomycin resistant; and UI, uninjected flies.

Therefore, we proceeded to test the virulence of our strains in *Drosophila*. Most (8/11) resistant strains showed a strong reduction in host killing in comparison with their paired susceptible strain (Fig. 1A). Only pairs J and K exhibited no difference in virulence between resistant and paired susceptible strains (Fig. 1A). The lack of difference in virulence between the strains in pair K was surprising since pairs A, C, G, I, and K differ by the same L826F amino acid substitution in MprF (Table 1). The different consequences observed in strains carrying the same mutation may be a result of the differences in genetic background among the strains.

The observation that all but three of the daptomycin-resistant strains exhibited a clear attenuation of virulence relative to their matched susceptible isolate suggests a fundamental link between daptomycin resistance and *Staphylococcus aureus* pathogenesis.

We next determined whether these differences in virulence reflected differences in the ability of the bacteria to grow in the host. This was assayed by quantifying viable bacterial counts in infected animals. We found that increased host mortality correlated with elevated viable bacterial numbers 18–20 h after infection (Fig. 1B). In most cases, the fly was able to control the growth of daptomycin-resistant strains but was unable to control the paired daptomycin-susceptible strain. This was not a general proliferation defect since the paired resistant and susceptible strains did not differ in growth rates in four different laboratory media (Fig. S1).

**Fig 2:**
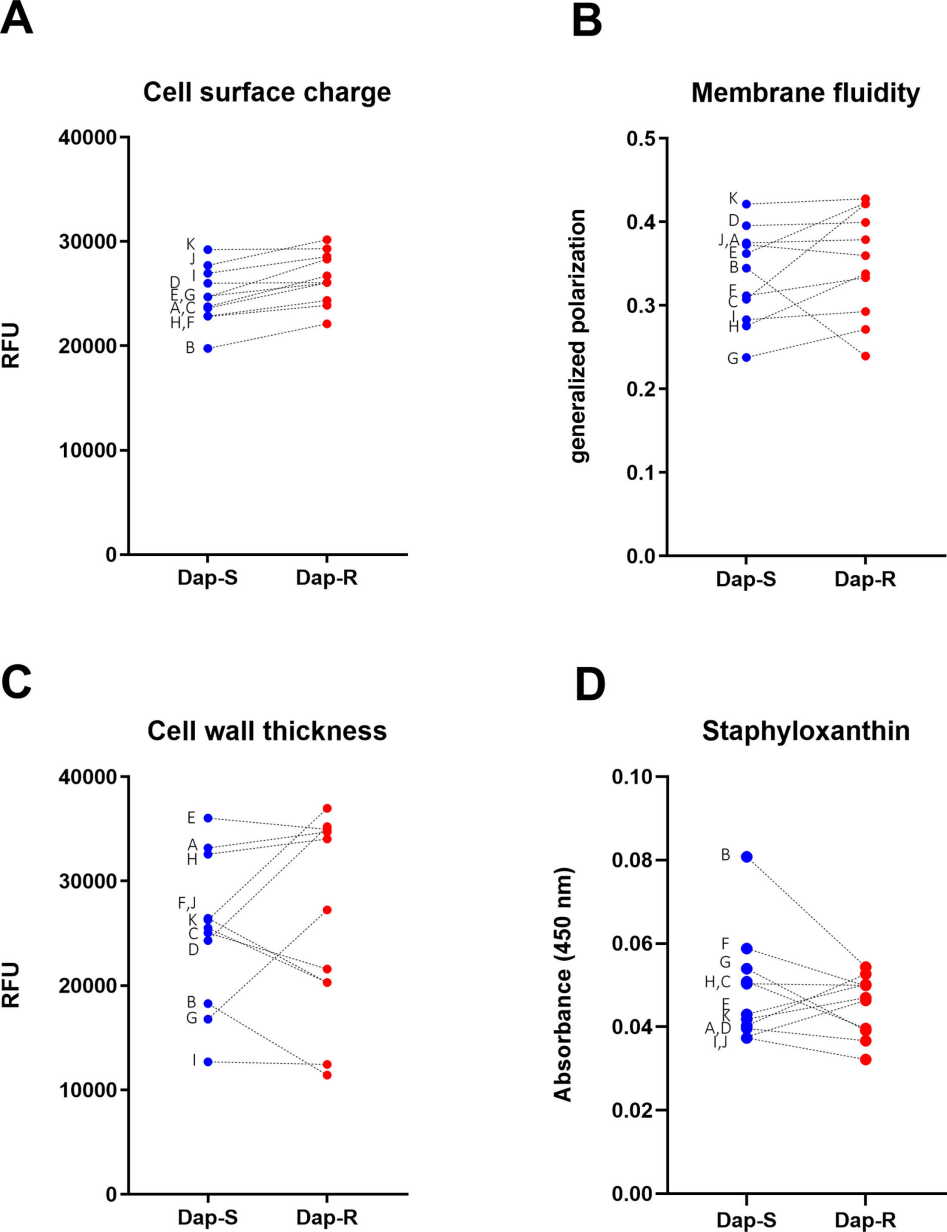
Cell surface properties do not correlate with daptomycin resistance or virulence. Data from three independent experiments. Dashed lines link paired strains; letters indicate pair name. Plots represent mean. Error bars are omitted for clarity. (**A**) Cell surface charge, *P* = 0.004, see also Fig. S2 for detailed results; (**B**) fluidity of cell membrane, *P* > 0.05, see also Fig. S3 for detailed results; (**C**) cell wall thickness, *P* > 0.05, see also Fig. S4 for detailed results; (**D**) cell surface carotenoid content, *P* > 0.05, see also Fig. S5 for detailed results. Data were analyzed by a two-tailed paired Student’s *t*-test. Dap-S, daptomycin susceptible; Dap-R, daptomycin resistant; and RFUs, relative fluorescence units.

Daptomycin resistance is frequently associated with changes in various cell surface characteristics (45). We examined several cell envelope properties of the isolates, including membrane fluidity, cell surface charge, and cell wall thickness. We also examined the production of the carotenoid pigment staphyloxanthin, as this has been shown to confer protection against host defenses such as reactive oxygen species. However, there were no significant differences between daptomycin-susceptible and resistant strains for any of these properties (Fig. 2; Fig. S2 to S5).

### Reduced virulence of daptomycin-resistant isolates is not due to altered virulence factor production

*S. aureus* produces a wide range of different virulence factors, including several cytolytic toxins expressed under the control of the Agr quorum-sensing system (46). Since toxin production is important for both human and *Drosophila* infection (46–49), we hypothesized that the lack of virulence of daptomycin-resistant isolates could be due to reduced virulence factor production.

**Fig 3:**
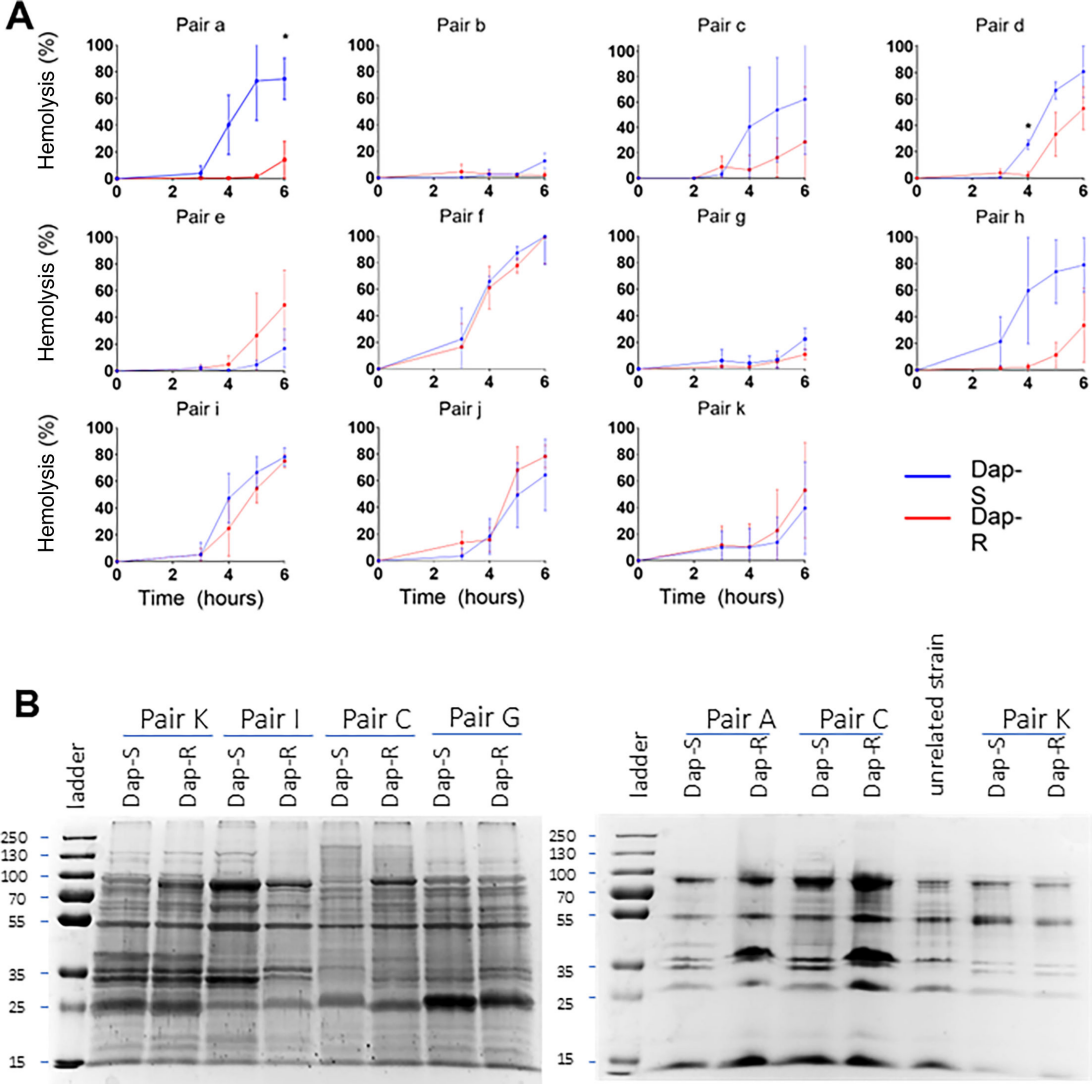
Virulence changes are not driven by changes in toxin secretion. (**A**) Hemolytic activity of culture supernatants from all paired strains. Blood incubated in tryptic soy broth (TSB) as negative control; TSB containing 0.1% Triton X-100 as positive control, considered to represent 100% lysis. Error bars, where shown, represent the standard deviation of the mean. Data were analyzed by a two-way ANOVA with Sidak’s *post hoc* tests. *P*-values for the difference in hemolysis activity produced by Dap-S vs Dap-R. *P*-values < 0.05 are indicated with *. Pair A, *P* = 0.0375 (6 h); pair D, *P* = 0.0058 (4 h); all other *P*-values > 0.05. (**B**) 10% SDS-PAGE of 10-fold concentrated supernatants from 4 h cultures. All pairs shown on this gel exhibit the same MprF amino acid substitution (L826F). Gel representative of two independent assays. Dap-S, daptomycin susceptible; Dap-R, daptomycin resistant.

To test this, we assessed the production of exotoxins by quantifying hemolytic activity as this is a marker of activity of the Agr quorum-sensing system (20). Most strains exhibited hemolytic activity after 4 h growth in broth, consistent with the secretion of hemolytic toxins resulting from Agr activity. While hemolytic activity varied among pairs, we did not detect a consistent difference between individual isolates within pairs (Fig. 3A). We then extended this analysis to proteins secreted by the bacterial cells (the exoproteome), focusing on pairs that carried the same MprF amino acid substitution (L826F, pairs A, C, G, I, and K). Again, we found variation in SDS-PAGE profile among pairs, but no consistent difference between individual isolates within pairs (Fig. 3B). Collectively, these data suggest that reduced virulence exhibited by daptomycin-resistant strains is not related to differences in exotoxin production.

### Reduced virulence of daptomycin-resistant isolates is not due to increased immune activation

In *Drosophila,* the Toll signaling pathway and the antibacterial melanization response rely on a common peptidoglycan-sensing mechanism (29). The melanization response is the primary immune effector mechanism functional against *S. aureus*, but the production of antimicrobial peptides driven by Toll pathway activation also contributes to host defense against this bacterium, and the expression of peptide genes is a well-established marker of Toll activation (29).

**Fig 4:**
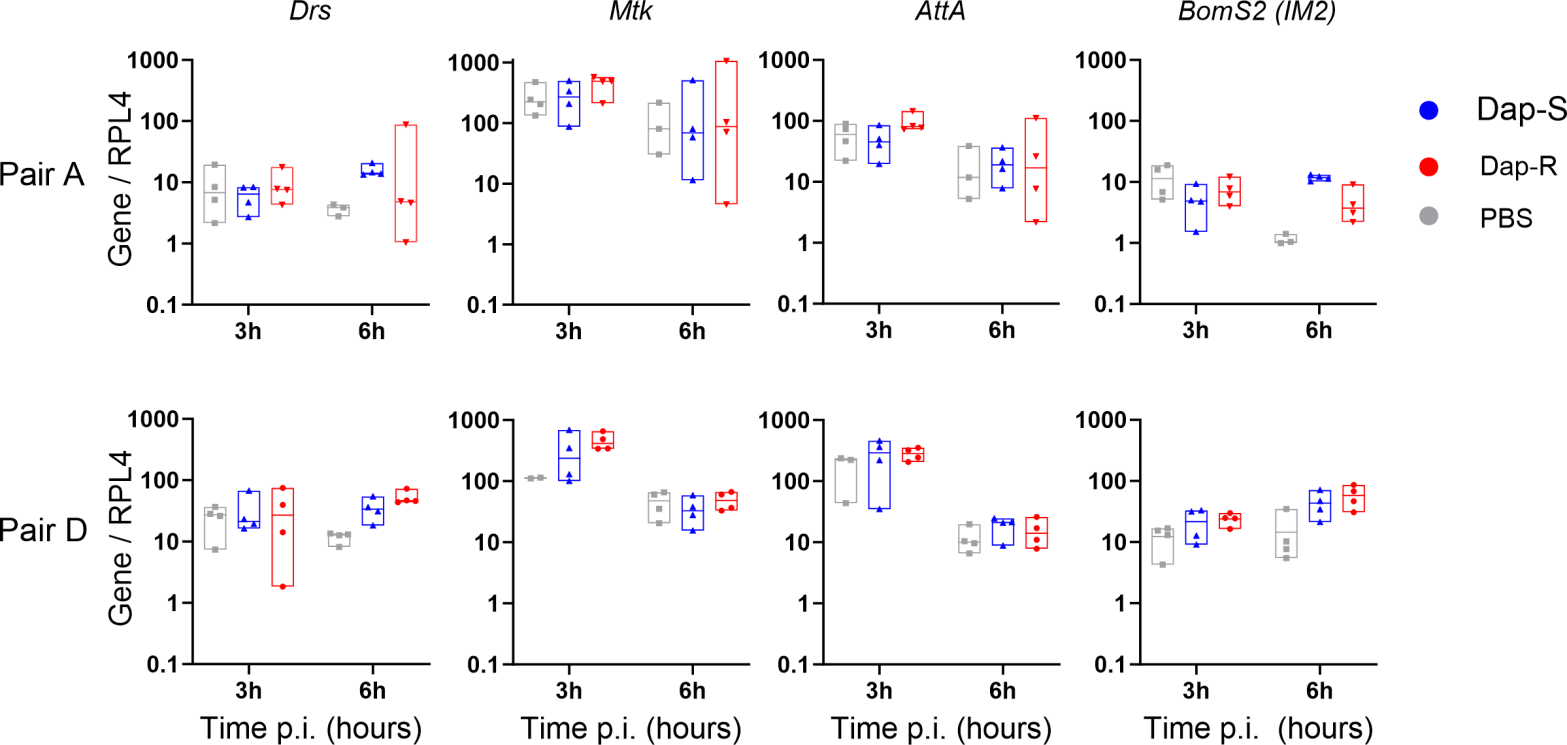
AMP genes are expressed at similar levels following infection with daptomycin-resistant and susceptible strains. Host AMP gene expression at 3 and 6 h after fly infection. Assessed AMPs are representative of immune pathways activated by bacterial infection. Expression levels of AMPs are shown normalized to *Rpl4* and then to the mean value for uninjected flies. Boxplots represent the median and percentiles 25 and 75. Data were analyzed by ANOVA followed by Tukey’s *post hoc* test for multiple comparisons. *Drs*, pair A, 3 h: *P* > 0.05 for all comparisons; 6 h: *P* > 0.05 for all comparisons; pair D, 3 h: *P* > 0.05 for all comparisons; 6 h: Dap-S and PBS, *P* = 0.0523; Dap-R and PBS, *P* = 0.024; Dap-S and Dap-R, *P* > 0.05. *Mtk* and *AttA*, 3 and 6 h: *P* > 0.05 for all comparisons (all pairs). *BomS2*: pair A, 3 h: *P* > 0.05 for all comparisons; 6 h: Dap-S and PBS, *P* = 0.0004; Dap-R and PBS, *P* > 0.05; Dap-S and Dap-R, *P* = 0.0032; pair D, 3 h: *P* > 0.05 for all comparisons; 6 h: Dap-S and PBS, *P* > 0.05; Dap-R and PBS, *P* = 0.0341; Dap-S and Dap-R, *P* > 0.05. Dap-S, daptomycin susceptible; Dap-R, daptomycin resistant.

To test whether daptomycin-susceptible and resistant strains elicit different levels of immune response activation, we measured the expression of genes in response to immune activation for strain pairs A and D. As expected, PBS injection induced *Drosomycin* (*Drs*), *Metchnikowin* (*Mtk*), *Attacin A* (*AttA*), and *Bomanin S2* (*BomS2*) expression (Fig. 4). Daptomycin-susceptible and resistant isolates induced the expression of these peptides at levels similar to or higher than PBS injection. There was no strong difference between daptomycin-susceptible and resistant isolates in the expression of any peptide genes tested. These results support the idea that the difference in virulence between paired isolates is not derived from differential ability to evade detection by the mechanisms upstream of Toll activation and melanization.

It remained possible that the daptomycin-susceptible strains could interfere with some aspect of the immune response other than antimicrobial peptide transcription. In this case, co-infection with daptomycin-susceptible and resistant strains would allow daptomycin-resistant strains to grow *in vivo* to a similar degree to their paired susceptible strain. Therefore, we co-infected *Drosophila* with 1:1 mixtures of susceptible and resistant bacteria, and we measured the numbers of viable susceptible and resistant bacteria 20 h after infection. This was explored for pairs A and D.

**Fig 5:**
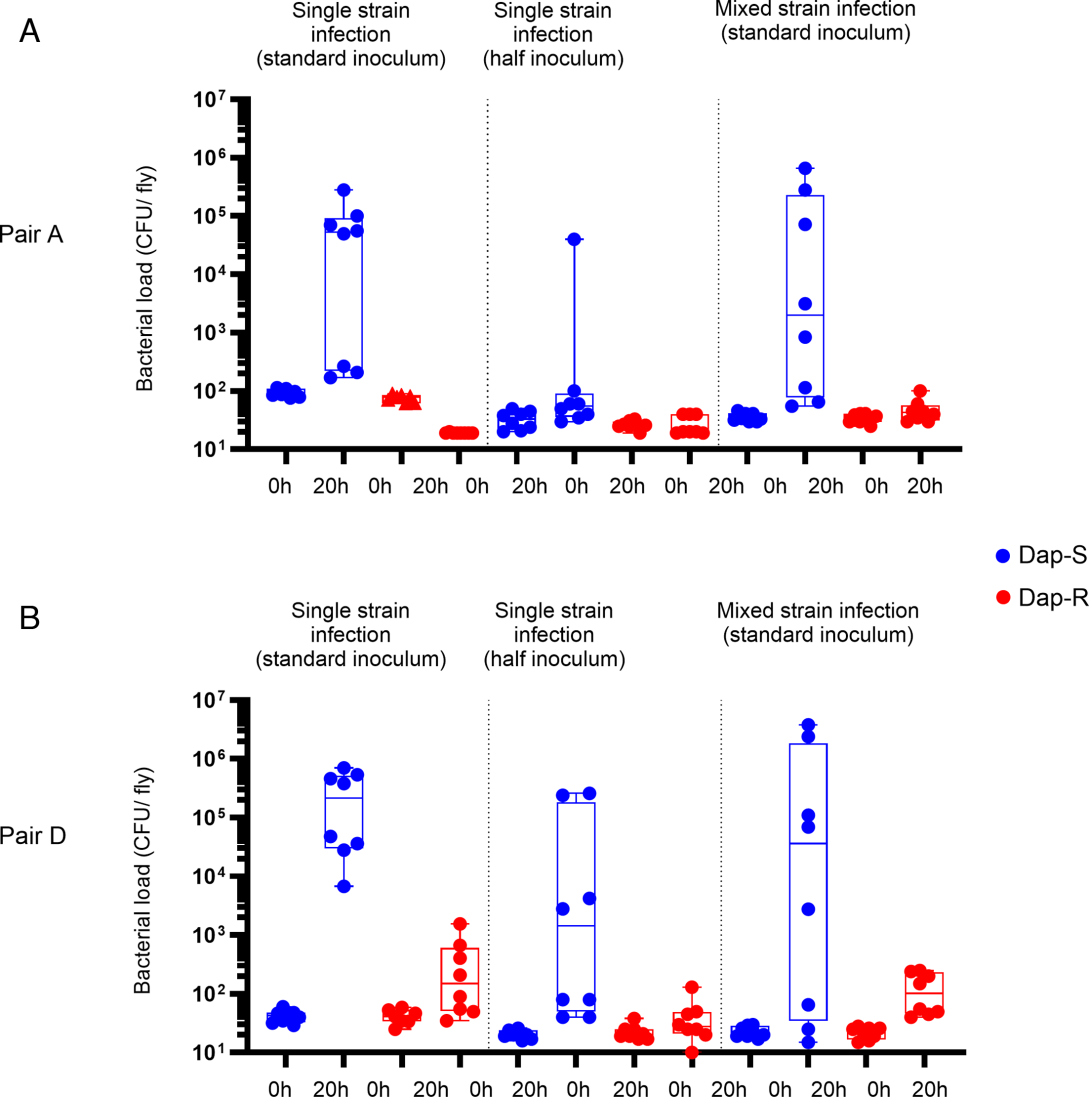
Daptomycin-susceptible strains do not confer protection on co-infected daptomycin-resistant bacteria. Strain colony counts at 0 h (input inoculum) and 20 h after injection of wild-type (*w^1118^*) flies with Dap-S strain, Dap-R paired strain, or Dap-S strain mixed with Dap-R paired strain. The ratio Dap-S:Dap-R in the mixed condition is 1:1; the total inoculum in co-infection experiments is the same as the standard inoculum used in other experiments. Bacterial counts presented correspond to one experiment representative of two independent experiments. Boxplots represent the median, percentiles 25 and 75, and whiskers to the minimum and maximum values. *P*-values for bacterial numbers at 20 h post-infection (Dap-R vs Dap-S paired strain) were calculated using a Kruskal-Wallis test with Dunn’s *post hoc* test for multiple comparisons. (**A**) Pair A, Dap-S/Dap-R co-infection assay. Single-strain infections were performed with an average input inoculum of 92 colony-forming unit (CFU)/fly (Dap-S) and 75 CFU/fly (Dap-R, standard inoculum) and of 26 CFU/fly (Dap-R) and 33 CFU/fly (Dap-S, half inoculum). Mixed strain infections were performed with an average of 36 CFU/fly (Dap-S) and 35 CFU/fly (Dap-R), which resulted in an average total inoculum of 71 CFU/fly. At 20 h, all *P*-values > 0.05, except: Dap-S vs Dap-R, standard inoculum: *P* = 0.05; Dap-S, half inoculum vs Dap-R, co-infection: *P* = 0.045. (**B**) Pair D, Dap-S/Dap-R co-infection assay. Single-strain infections were performed with an average input inoculum of 42 CFU/fly (Dap-S) and 37 CFU/fly (Dap-R, standard inoculum) and of 20 CFU/fly (Dap-S) and 22 CFU/fly (Dap-R, half inoculum). Mixed strain infections were performed with an average of 23 CFU/fly (Dap-S) and 22 CFU/fly (Dap-R), resulting in an average total inoculum of 45 CFU/fly. At 20 h, all *P*-values > 0.05, except Dap-S vs Dap-R, standard inoculum: *P* = 0.004; Dap-S vs Dap-R, half inoculum: *P* = 0.053; and Dap-S vs Dap-R, co-infection: *P* = 0.046.

As before, in single-strain infections, daptomycin-susceptible strains grew *in vivo*, while daptomycin-resistant bacteria did not. In co-infection experiments, the presence of daptomycin-susceptible bacteria was not able to rescue the ability of daptomycin-resistant bacteria to grow *in vivo* (Fig. 5A and B).

Taken together, these data indicated that the reduced virulence of daptomycin-resistant isolates relative to their matched daptomycin-susceptible partner was not due to enhanced immune activation. Alternatively, these results could still reflect competition between the daptomycin-resistant and the sensitive parental strain.

### Daptomycin-resistant strains are more susceptible to killing by melanization but not to antimicrobial peptides

**Fig 6:**
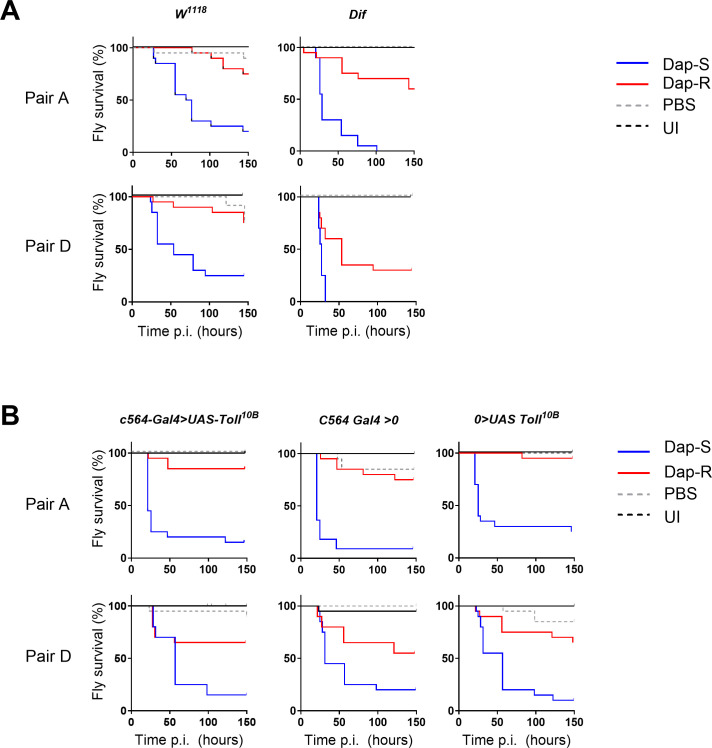
Daptomycin resistance does not increase sensitivity to Toll-dependent antimicrobial effectors. Data presented for two pairs, pair A and pair D. (**A**) Survival of wild-type (*w^1118^*) and *Dif*-mutant flies infected with Dap-S or Dap-R paired strains. Survival curves presented correspond to one assay representative of at least two independent experiments with similar results. (**B**) Survival of control (*C564-Gal4 >* 0 and 0 *> UAS-Toll^10B^*) and *Toll^10b^*-expressing (*C564-Gal4 > UAS-Toll^10B^*) flies infected with Dap-S and Dap-R paired strains. Survival curves correspond to one assay representative of two independent experiments.

Since daptomycin-susceptible strains were unable to protect resistant strains from host immune defenses, we assumed that susceptible strains were more resistant to host immune killing mechanisms than resistant isolates (Fig. 4 and 5). To test this, we first investigated whether Toll-dependent antimicrobial peptides could be responsible for this difference by measuring survival after infection of *Dif*-mutant flies, which lack almost all Toll-dependent inducible gene expression, and Bomanin-deficient flies, which lack a family of peptides important in defense against some gram-positive bacteria (50, 51). However, the absence of *Dif* or Bomanins did not affect the survival difference between paired susceptible and resistant strains (Fig. 6A; Fig. S6). We then infected transgenic flies expressing *Toll^10B^*, a constitutive allele of *Toll* (52). As had been seen in wild-type flies, daptomycin-susceptible strains rapidly killed flies with constitutively active Toll signaling, while their resistant counterparts were significantly attenuated (Fig. 6B). Taken together, these results indicate that daptomycin resistance-related attenuated virulence is not associated with increased sensitivity to Toll-dependent antimicrobial peptides or Bomanins.

**Fig 7:**
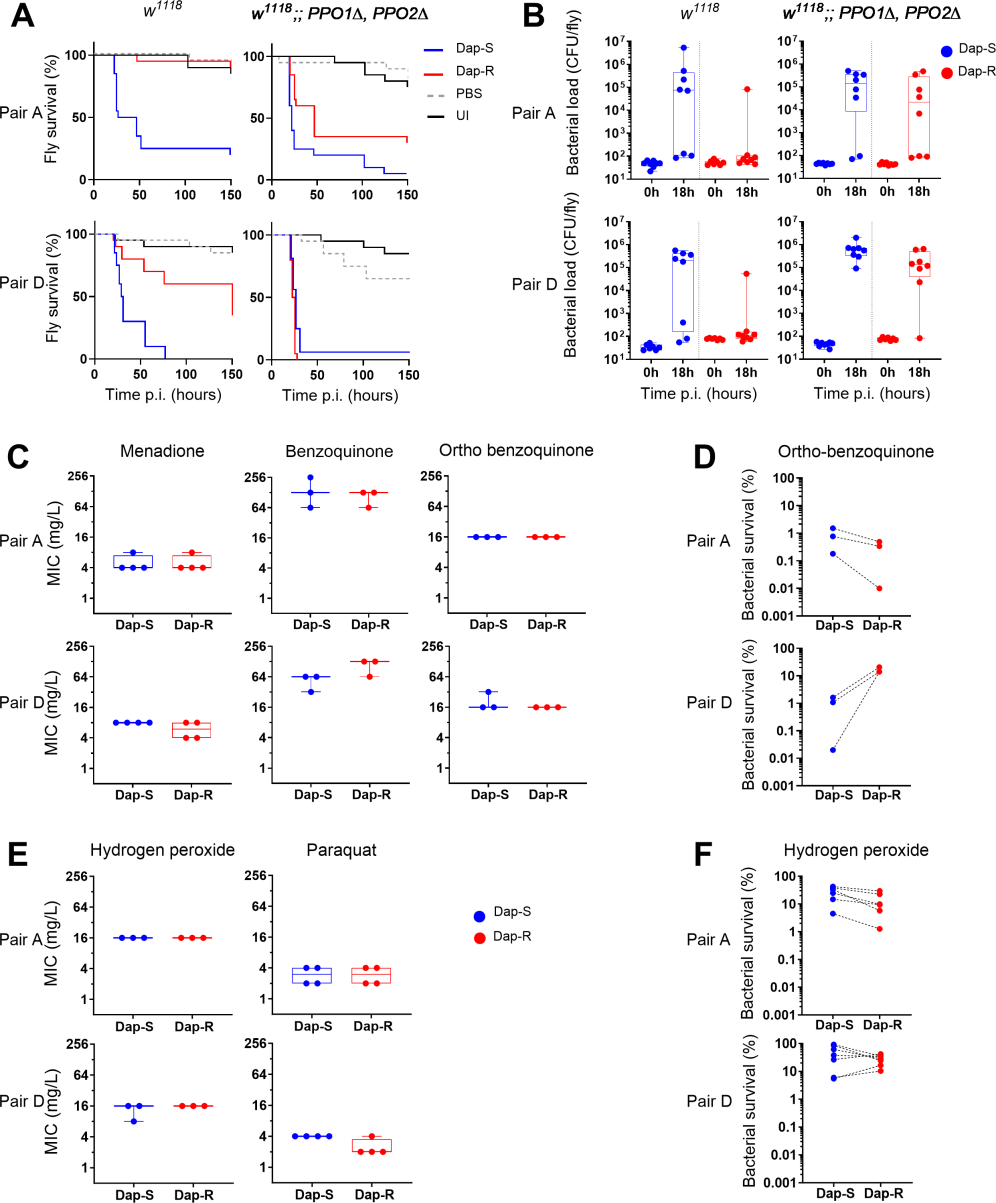
Dap-R staphylococcal virulence is affected by prophenoloxidase but cells are not more susceptible to quinones and reactive oxygen species. Data presented for 2 pairs, pair A and pair D. (A) Survival of wild type (w^1118^) and w^1118^; PPO1^Δ^ PPO2^Δ^ flies infected with either Dap-S strain or Dap-R paired strain. Survival analysis reveals similar mortality of PPO1^Δ^ PPO2^Δ^ double mutant flies whether infected with Dap-S or Dap-R strains. Survival curves presented correspond to one assay representative of at least two independent experiments with similar results. Survival curves presented correspond to one assay representative of at least two independent experiments with similar results. See also Fig. S7A for other pairs. (B) Bacterial numbers at 0 h (input inoculum) and 18 h after injection of wild type (w^1118^) or w^1118^; PPO1^Δ^ PPO2^Δ^ flies with Dap-S strain and Dap-R paired strain. Counts from one assay representative of at least two independent experiments. Bacterial load is similar Dap-R and Dap-S isolates in PPO1^Δ^ PPO2^Δ^ flies (pair A, median bacterial numbers at 18h : in w^1118^ , Dap-S, 7.6x10^5^ CFU/fly vs Dap-R, 72 CFU/fly; in PPO1^Δ^ PPO2^Δ^, Dap-S, 1.4 x10^5^ CFU/fly vs Dap-R, 2.1 x10^4^ CFU/fly; Mann-Whitney test, *P* = 0.0104 (w^1118^), *P* = 0.4609 (PPO1^Δ^ PPO2^Δ^); pair D, median bacterial numbers at 18h in w^1118^: Dap-S, 2.1 x10^5^ CFU/fly vs Dap-R, 112 CFU/fly; in PPO1^Δ^ PPO2^Δ^: Dap-S, 6.0 x10^5^ CFU/fly vs Dap-R, 1.3 x10^5^ CFU/fly; Mann-Whitney test, *P* =0.0779 (w^1118^), *P* = 0.0379 (PPO1^Δ^ PPO2^Δ^). CFU, colony forming unit. (C) Minimum inhibitory concentration of three quinones for Dap-S and Dap-R paired strains of pair A and pair D. No growth difference between Dap-R and Dap-S paired strains in presence of any of three tested quinones (menadione, benzoquinone, ortho-benzoquinone) (two-tailed paired Student’s *t*-test except for pair A/orthobenzoquinone, (Mann-Whitney test), *P* > 0.05 for all pairs). (D) In vitro survival of Dap-S and Dap-R paired strain of two pairs exposed to ortho-benzoquinone (16 mg/L) for 1 h, as determined by CFU counts. Two-tailed paired Student’s *t*-test, ortho-benzoquinone: pair A, *P* = 0.1665; pair D, *P* = 0.0144. (E) Minimum inhibitory concentration of two reactive oxygen species (ROS) for Dap-S and Dap-R paired strains of pair A and pair D. No growth difference between Dap-R and Dap-S paired strains in presence of any of two reactive oxygen species (hydrogen peroxide, paraquat) (two-tailed paired Student’s *t*-test except for pair A/ hydrogen peroxide and paraquat (Mann-Whitney test), *P* > 0.05 for all pairs). (F) In vitro survival of Dap-S and Dap-R paired strain of two pairs exposed to hydrogen peroxide (4 mM) for 1 h, as determined by CFU counts. Two-tailed paired Student’s *t*-test, hydrogen peroxide: pair A, *P* = 0.0127; pair D, *P* = 0.1947.

We next investigated whether differential sensitivity to melanization was responsible for the virulence difference between daptomycin-susceptible and resistant strains. We tested this by determining the sensitivity of flies lacking the prophenoloxidases *PPO1* and *PPO2*; these enzymes are required for immune-induced melanization and are important for defense against *S. aureus* (53). We observed that the difference in survival between flies infected with daptomycin-susceptible and resistant paired strains was consistently reduced in *PPO1^Δ^*, *PPO2^Δ^* double mutants than in wild-type controls (Fig. 7A; Fig. S7A). This effect was also seen in bacterial loads: daptomycin-susceptible strains grew to equal numbers in wild-type and *PPO1^Δ^, PPO2^Δ^* flies, while daptomycin-resistant strains exhibited little growth in wild-type flies but grew in *PPO1^Δ^, PPO2^Δ^* flies to similar numbers as susceptible strains (Fig. 7B). These findings demonstrate that the daptomycin resistance-related loss of virulence results from differential sensitivity to killing by phenoloxidase.

The mechanism by which phenoloxidase kills bacteria is not entirely clear; in addition to melanin, this reaction produces reactive oxygen and cytotoxic quinones as intermediates (30, 31, 54). Phenoloxidases are thought to generate direct antimicrobial activity through these toxic products (54). We thus investigated whether the observed differential sensitivity to melanization could be attributed to differential susceptibility to either reactive oxygen species or quinones. Because the quinones generated during the melanization reaction (e.g., dopaquinone) are highly labile and thus not commercially available, we investigated this question by assaying sensitivity *in vitro* to menadione, para-benzoquinone, and ortho-benzoquinone and to two reactive oxygen species (hydrogen peroxide and paraquat). We found no difference in minimum inhibitory concentration of any of these compounds (Fig. 7C). Ortho-quinones, those produced by the melanization reaction, are normally considered the most reactive, so we further tested bacterial survival to ortho-benzoquinone. However, different pairs exhibited different changes in sensitivity to killing by ortho-benzoquinone, indicating that sensitivity to quinones is unlikely to represent the common mechanism driving loss of virulence in Dap-R strains (Fig. 7D; Fig. S7B). Collectively, our data demonstrate that the loss of virulence in daptomycin-resistant strains reflects increased sensitivity to prophenoloxidase-dependent melanization *in vivo*.

## DISCUSSION

When subjected to antimicrobial selective pressure, *S. aureus* acquires resistance, which can affect its pathogenicity (19, 55). Using a *Drosophila* systemic infection model and a panel of paired clinical strains collected before and after daptomycin resistance acquisition, the largest so far reported, we show that acquisition of daptomycin resistance, most frequently through *mprF* mutation, is associated with attenuated pathogenicity that is mostly explained by greater susceptibility to *Drosophila* phenoloxidase activity. We also show that this attenuated virulence is independent of host detection via the Toll pathway, and that the virulence difference between daptomycin-resistant and susceptible strains is not a consequence of interference with host defense peptide expression. Finally, we show that this acquired susceptibility to phenoloxidase activity is not a consequence of a general sensitivity to quinones but does require direct contact between *S. aureus* and phenoloxidase.

Most of our daptomycin-resistant isolates differed from their daptomycin-sensitive parental strains by the presence of a single mutation of unknown functional impact in *mprF*. Mutations that confer daptomycin resistance have been assumed to lead to *mprF* gain-of-function, resulting in increased abundance of lysyl-phosphatidylglycerol in the outer leaflet of the membrane (56–58). This change would be expected to result in increased membrane charge, which might help repel cationic AMPs from the membrane, modulate the interaction of peptides with the membrane, or attenuate membrane perturbation by daptomycin and potentially other antimicrobial peptides (10, 59, 60). However, consistent with charge-mediated and -unrelated mechanisms involved in daptomycin resistance, we found no change in surface charge or in a range of other bacterial surface properties in our Dap-R strains (38, 41, 42, 57, 61). The reasons for this apparent difference are poorly understood; they may be related to some unexamined difference in genetic background or subtleties of the precise mutations identified. MprF is also believed to influence cell wall metabolism, membrane fluidity, membrane proteome, preformed toxin release, lipoteichoic acid length, and general fitness (34, 58, 62–64). Some combination of these effects enables MprF to contribute to commensal persistence during inflammation by conferring resistance to AMPs (64). Resistance to daptomycin may also result from an effect of MprF enzymatic activities other than those known (synthesis and transport of lysyl-phosphatidylglycerol) (65). Finally, they may reflect some fundamental misunderstanding of the interaction between daptomycin and MprF itself.

Despite this gap in our knowledge, what is clear is that *mprF*-related daptomycin resistance exhibits phenotypic crosstalk with staphylococcal pathogenic potential. Recently, it has been shown that the blockade of the MprF flippase activity sensitized MRSA to host AMPs and daptomycin, which interfered with pathogenicity in humans (66). Here, we found that *mprF* mutations, while conferring daptomycin resistance, also caused a decrease in virulence by increasing sensitivity to *Drosophila* host defense. The so-called “see-saw effect” refers to the observation that increased resistance to daptomycin is associated with reduced resistance to β-lactam antibiotics (39); our findings show a similar “see-saw” between daptomycin resistance and pathogenic potential.

When we initiated this work, we first wished to assess whether daptomycin resistance could compromise fly AMP action, in line with previous reports of cross-resistance between daptomycin and cationic AMP such as human LL-37 or hNP-1 (26, 42). Neither the strong daptomycin-resistant-associated attenuation of virulence nor the Toll signaling pathway-independent effect supports the concept of cross-resistance between daptomycin and host AMPs (Fig. 1A and 2A; Fig. S2). *Drosophila* host defense against *S. aureus* is dependent on the activities of many antimicrobial effectors, including antimicrobial peptides, Bomanins (a family of small, secreted peptides of unknown biochemical activity that can confer resistance to infection [51]), and phenoloxidase (29, 53, 67). Despite this wealth of effectors, our data clearly indicate that daptomycin resistance specifically increases susceptibility to killing via phenoloxidase. Phenoloxidase is the central activity in the melanization reaction, one of the most immediate immune responses in the fly; it catalyzes the oxidation of phenols (e.g., tyrosine) to quinones that spontaneously polymerize to form melanin (31). While much is known about PPO (e.g., location of PPO storage inside the cell, PPO 3D structure [54, 68, 69], the cascade by which PPO is activated into phenoloxidase [29], and the steps of the melanization reaction [31]), little is known about how phenoloxidase kills bacteria. One prominent view is that phenoloxidase activity generates highly reactive quinones, and these quinones are the relevant antimicrobial activity (70). Our daptomycin-resistant isolates do not generally exhibit increased sensitivity to quinones or ROS *in vitro*, but we were unable to directly test sensitivity to the specific quinones produced *in vivo* and thus cannot exclude increased sensitivity to these compounds in the host context.

Although we are unable to define the precise killing mechanisms of phenoloxidase, our observations have implications regarding how bacteria evade phenoloxidase-mediated killing. MprF has a well-defined role in modulating the staphylococcal cell surface. Here, we show that antistaphylococcal phenoloxidase activity is altered by the changes associated with MprF-related daptomycin resistance. Our data indicate that the cell-surface changes made by daptomycin-associated *mprF* alleles result in dramatically increased sensitivity to the bactericidal activity of phenoloxidase. Jearaphunt et al. (71) have demonstrated that agglutination accompanies, and may be the primary mechanism of, phenoloxidase-mediated killing. It is possible that the effect of daptomycin resistance-associated *mprF* alleles is either to enable the association between circulating phenoloxidase and the bacterial surface or the interaction between individual bacterial cells.

In a broader sense, our series of daptomycin-resistant clinical paired isolates exhibits a strong and nearly constant trend of daptomycin resistance-associated virulence attenuation *in vivo*, consistent with previous observations in mouse, *Galleria*, and zebrafish infection models (26, 35), even though this see-saw effect requires further study in other hosts. The fact that daptomycin resistance is associated with reduced virulence in hosts with different immune systems, some of which lack antimicrobial phenoloxidase (e.g., zebrafish and mouse), is intriguing. One possibility is that this general effect is associated with a general difference in the tendency to agglutinate. Another possibility is that the cell-surface modifications present in *mprF*-associated daptomycin resistance result in increased affinity for many immune effectors. Identification of the cell-surface properties responsible for this differential immune susceptibility is potentially a new staphylococcal Achilles heel. Finally, some assayed hosts (e.g., zebrafish and mouse) lack antimicrobial phenoloxidase, but they all bear some tyrosinases, and this may suggest a role of tyrosinases in this effect to be further explored.

## MATERIALS AND METHODS

### Bacterial strains and culture conditions

*S. aureus* strains used in this study are listed in Table S1. Strains were grown overnight in tryptic soy broth (TSB) unless specified, at 37°C with shaking (180 rpm) (72). Where required, medium was supplemented with erythromycin (10 µg/mL). Enumeration of CFU was performed by culturing 10-fold serial dilutions of samples onto tryptic soy agar (TSA) plates. Where required (competition assay), TSA used for enumerations was supplemented with daptomycin at an appropriate concentration and 1.25 mM CaCl_2_.

### Bacterial growth assay

Stationary-phase bacteria were diluted 1,000-fold in fresh broth into a 96-well plate. The plate was incubated in a TECAN Infinite 200 PRO microplate reader. Bacterial growth was monitored using measurements of optical density at 600  nm (OD_600 nm_). Bacterial growth was assayed in TSB, cation-adjusted Muller-Hinton Broth (MHB), Dulbecco’s Modified Eagle Medium supplemented with 1% amino acids except for pair A strains (5% amino acids) and RPMI 1640.

### Genome sequencing and genome analysis

Whole genome sequencing was performed using Illumina technologies, as previously described (73). Assemblies were obtained with SPAdes (74). The presence of point mutations associated with daptomycin was analyzed using Point Finder and an in-house curated database. Mutations were corroborated by single nucleotide polymorphism detection performed for each paired isolates using Prokka (75) and Snippy (https://github.com/tseemann/snippy).

### Determination of MICs

MICs were determined using a twofold serial broth dilution protocol in cation-adjusted MHB, as previously described (76). MHB was supplemented with either 1.25 mM CaCl_2_ or 2% NaCl for the determination of daptomycin and oxacillin MICs, respectively. Hydrogen peroxide, menadione, benzoquinone, or ortho-benzoquinone MICs were determined with freshly prepared solutions. The MIC was defined as the lowest concentration at which no visible growth was observed.

### Determination of bacterial survival to ortho-benzoquinone and hydrogen peroxide

As described elsewhere (76), TSB-grown cells were washed in PBS. A total of 10^4^ cells (10 µL) was mixed with 90 µL of freshly diluted ortho-benzoquinone (16 mg/L in PBS), freshly diluted hydrogen peroxide (4 mM/L in PBS), or PBS (for 0 h time point) in 96-well plates. Bacterial survival was determined after 1 h at 37°C in the dark without shaking, by enumerating CFU from 10-fold serial-diluted suspensions on agar plates. Results were expressed as a percentage of the number of bacteria in the initial inoculum.

### Determination of the hemolytic activity

Hemolysis of human erythrocytes was used to measure the hemolytic activity of *S. aureus* hemolysins, as described previously (77), with aliquots from serial twofold dilutions (100 µL) being mixed with an equal volume of 5% human blood suspension in PBS. Bacteria from TSB-grown cultures were sampled at various time points (0, 2, 3, 4, 5, and 6 h). The intensity of erythrocyte cell lysis was determined by measuring the absorbance of supernatants at 570 nm (A570) using an iMark microplate reader (Bio-Rad) (76, 77). The percentage of hemolysis for each sample and time point was calculated relative to the positive control (TSB containing 0.1% Triton X-100). All values used in calculations were related to the reading of undiluted supernatants from the bacterial cultures.

### Determination of *S. aureus* secreted proteins (exoproteome)

Bacteria were removed from 4 h spent culture medium by centrifugation (4,000 × *g*; 15 min). Thirty milliliters of 40% trichloroacetic acid in acetone was added to the supernatant (10 mL), and the tubes were incubated at −20°C overnight (78). Protein pellets were washed twice with acetone, concentrated 10-fold by centrifugation, and dried. Pellets were resuspended in sample electrophoresis treatment buffer. Samples were heated, centrifuged, and run on 10% SDS-polyacrylamide gels. The gels were stained for protein with Coomassie blue and destained before being imaged on a Gel Doc EZ imager (Bio-Rad).

### *Drosophila* stock and generation

Flies were maintained on a standard diet as detailed elsewhere (79). The fly lines used in this study are described in Table S2. For crosses, flies were sheltered at 25°C, apart from flies expressing Toll^10b^, which were housed at room temperature until eclosion. After hatching, male flies were collected and kept at 25°C on fly medium. Five- to eight-day-old male flies were used for all experiments. Unless specified otherwise, W^1118^ flies (hereafter wild-type flies) were used for experiments.

### *Drosophila* injections

Fly injections were performed as previously described (79), using a pulled-glass capillary needle and a Picospritzer injector instrument (Parker) to inject 50 nL of a standardized bacterial suspension in the abdomen of each fly. Control flies included flies injected with sterile PBS and uninjected flies. After inoculation, flies were housed at 29°C on a standard diet.

For bacterial injection, suspensions were prepared as follows: TSB-grown bacteria (15 h) were resuspended in sterile PBS (OD_600_ = 0.1) and further diluted 1:50, except for pair C (dilution 1:10). This resulted in an approximate average inoculum of 50–80 CFU in the fly body (hereafter standard inoculum). Infectious dose was checked by culturing the serially diluted bacterial suspensions and two individual flies homogenized instantly after injection in PBS (100 µL) and plated onto a TSA plate.

For competition assays, flies were injected with a mixture of the paired strains at a ratio of 1:1. This mixture comprised a total amount of bacteria corresponding to the standard inoculum, and half of that inoculum was daptomycin-susceptible strains, while the other half was daptomycin-resistant strains. Control flies included, in addition to the standard controls (PBS-injected and uninjected flies), flies injected with either individual strain at standard inoculum or individual strain at twofold diluted standard inoculum (half standard inoculum). These controls were designed to compare the outcomes of monomicrobial and mixed infections with either the same total amount of bacteria (all cells cumulated) or with the same amount of each individual microorganism.

### Fly survival assays

For each experiment, 20 adult flies per condition were injected, and survival was regularly assessed for 6 days by scoring dead flies. At least two independent experiments were carried out.

### Drosophila gene expression—RT-qPCR

Samples were collected 3 and 6 h after infection and processed as described elsewhere using a Corbett Rotor-Gene 6000 (80). Aliquot of every cDNA sample (10 µL) was 40-fold diluted before being used for gene quantification by qRT-PCR, using Sensimix with qPCR SyGreen 2× qPCR mix (PCR Biosystems). The cycling conditions were held at 95°C for 10 min, then 45 cycles of 95°C for 15 s, 57°C for 30 s, and 72°C for 30 s, followed by a melting curve. Primers used are listed in Table S3. The assay was performed twice with at least three biological replicates per condition per experiment.

### Bacterial quantification in the fly

At least 16 flies were injected with *S. aureus* daptomycin-susceptible strain or its resistant paired strain. From these, eight flies were collected for 0 h quantifications, while the remaining flies were housed at 29°C for 18–20 h before bacterial quantification. In any case, individual flies were homogenized in sterile PBS (100 µL). The entire homogenates were plated undiluted onto TSA for flies collected at 0 h, while samples from flies collected at 18–20 h were serially diluted and plated onto TSA plates, where they incubated for 16–18 h. The number of CFUs present in each fly was determined from the number of colonies following incubation. All quantifications were performed twice.

For the competition assay, the homogenates of flies infected with the mix (daptomycin-susceptible and resistant paired strains) were also plated onto TSA supplemented with 1.25 mM CaCl_2_ and daptomycin (hereafter Dap-TSA) to a final concentration of 0.5 mg/L (pair A strains) or 1 mg/L (pair D strains). Following incubation and colony counting, the number of daptomycin-resistant strain CFUs present in each fly was calculated from the number of colonies counted on the Dap-TSA plates, while the number of daptomycin-susceptible strain CFUs was determined from the difference between the number of colonies on TSA (daptomycin-susceptible and resistant strain CFUs) and on Dap-TSA plates (daptomycin-resistant strain CFUs). Because the homogenates (100 µL) of flies collected for bacterial quantification at 0 h were entirely spread onto plates, an extra eight flies were injected with the mix for quantifications at 0 h on Dap-TSA, as described above.

### Determination of membrane fluidity

The fluidity of the cell membrane was assayed by Laurdan generalized polarization with 500 µL stationary-phase culture (17 h) and 100 µM Laurdan, as detailed elsewhere (81, 82). A higher GP value indicated a relatively more rigid membrane, while a lower GP value indicated a more fluid membrane.

### Determination of peptidoglycan production

The cell wall thickness was assessed using HCC-amino-d-alanine (HADA), a fluorescent d-amino acid analog that is incorporated into peptidoglycan during its synthesis. As described elsewhere, bacteria were TSB-grown in the dark with 25 µM HADA (72). The samples were washed three times in PBS. HADA fluorescence intensities were measured using a Tecan Infinite 200 Pro plate reader (excitation 405 nm and emission 450 nm). Measurements generated values expressed as relative fluorescence units (RFUs), which correlate with the amount of peptidoglycan produced and, indirectly, with the cell wall thickness during growth.

### Determination of surface charge

For the measurement of bacterial surface charge, assays were performed as described elsewhere using 80 µg/mL fluorescein isothiocyanate FITC-labeled Poly-L-Lysine (FITC-PLL) and overnight TSB-grown bacteria (72, 83). After samples were washed three times, the fluorescence was measured using a TECAN Infinite PRO 200 plate reader (excitation 485 nm and emission 525 nm). A higher signal, expressed as RFU, reflected an increased FITC-PLL binding to the bacterial surface, which indicated a more negatively charged cell surface.

### Cell surface carotenoid extraction and quantification

The amounts of cell surface carotenoid content (including staphyloxanthin) were determined as previously described (76) using cells overnight TSB-grown and iMark microplate reader (Bio-Rad). Measurements at 450 nm estimate the amount of extracted staphyloxanthin.

### Statistical analysis

Experiments were performed on at least three independent occasions, unless specified otherwise. Data were analyzed by two-tailed Student’s *t* test or Mann-Whitney test (single comparisons) and ANOVA or Kruskal-Wallis test (multiple comparisons), when appropriate. Survival curves were plotted using the Kaplan-Meier method, and data were analyzed using the log-rank test. When appropriate, *post hoc* analysis (Tukey’s, Sidak’s, and Dunn’s) was performed to identify specific differences between conditions. A *P*-value of <0.05 was considered to reflect significance (GraphPad Prism).

## Data Availability

The sequence data have been deposited in the European Nucleotide Archive (ENA) at EMBL-EBI under accession numbers PRJEB72842 and PRJEB54647 for sample ERS12426074 and are listed in Table S1.

## SUPPLEMENTAL MATERIAL

Supplemental figures iai.00594-24-s0001.pdf Fig. S1 to S7.

Supplemental tables iai.00594-24-s0002.docx Tables S1 to S3.

ASM does not own the copyrights to Supplemental Material that may be linked to, or accessed through, an article. The authors have granted ASM a non-exclusive, world-wide license to publish the Supplemental Material files. Please contact the corresponding author directly for reuse.
