# Supplementary material for: Acquisition of daptomycin resistance in patients results in decreased virulence in Drosophila
Source: Infect Immun. 2025 May 23;93(6):e00594-24. doi: 10.1128/iai.00594-24 (PMC12150764; doi:10.1128/iai.00594-24)
Supplement: Supplemental figures — Fig. S1 to S7. [file iai.00594-24-s0001.pdf]

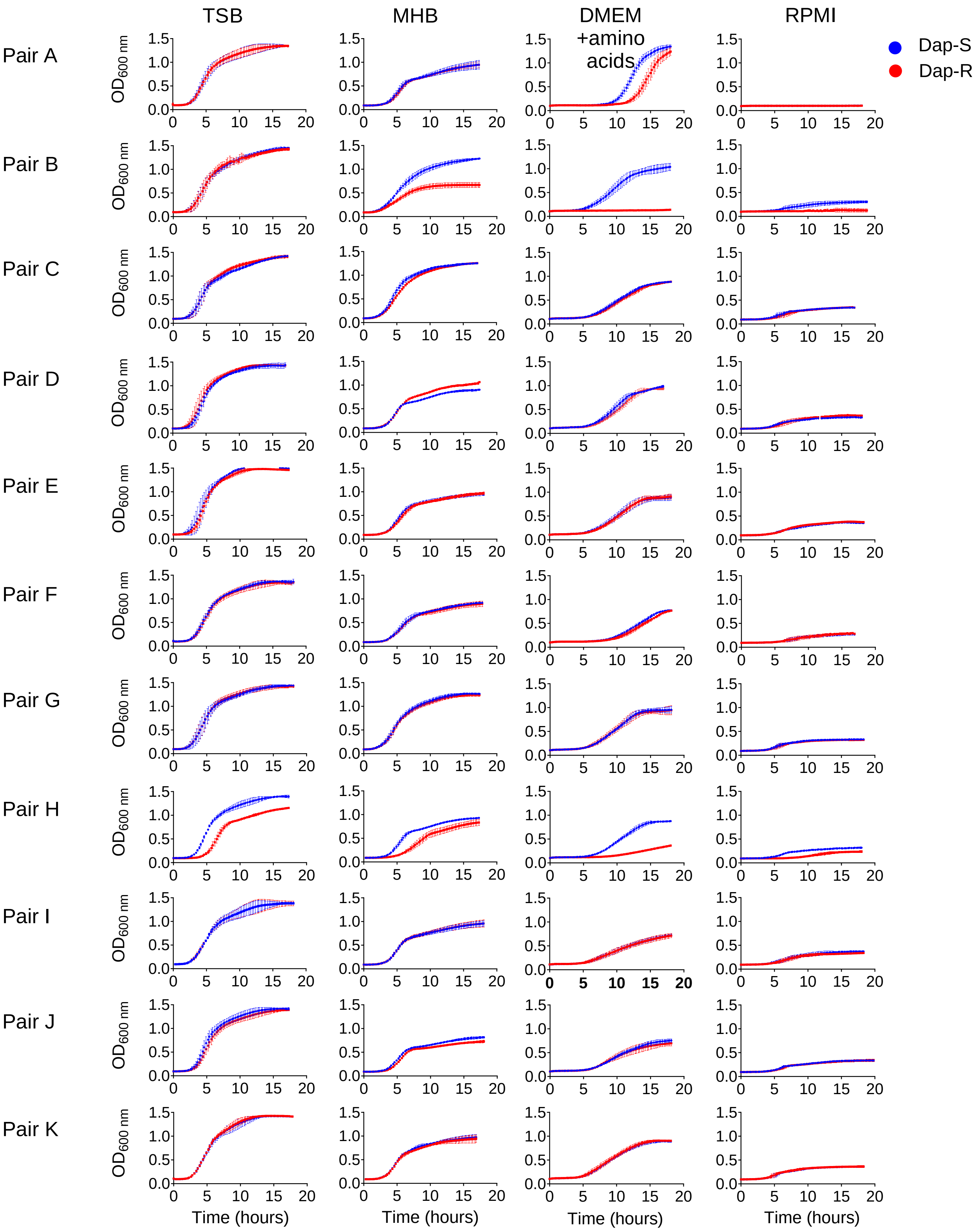

S1 Fig. Paired strains *in vitro* growth over time. Growth in (A) tryptic soy broth; (B) Muller Hinton broth; (C) Dulbecco's Modified Eagle Medium (DMEM) supplemented with amino acids; (D) RPMI 1640. Bacteria were grown at 37°C with shaking (700rpm), and In all cases, at least 3 replicates in duplicate or triplicate. Error bars represent the standard deviation of the mean.

.

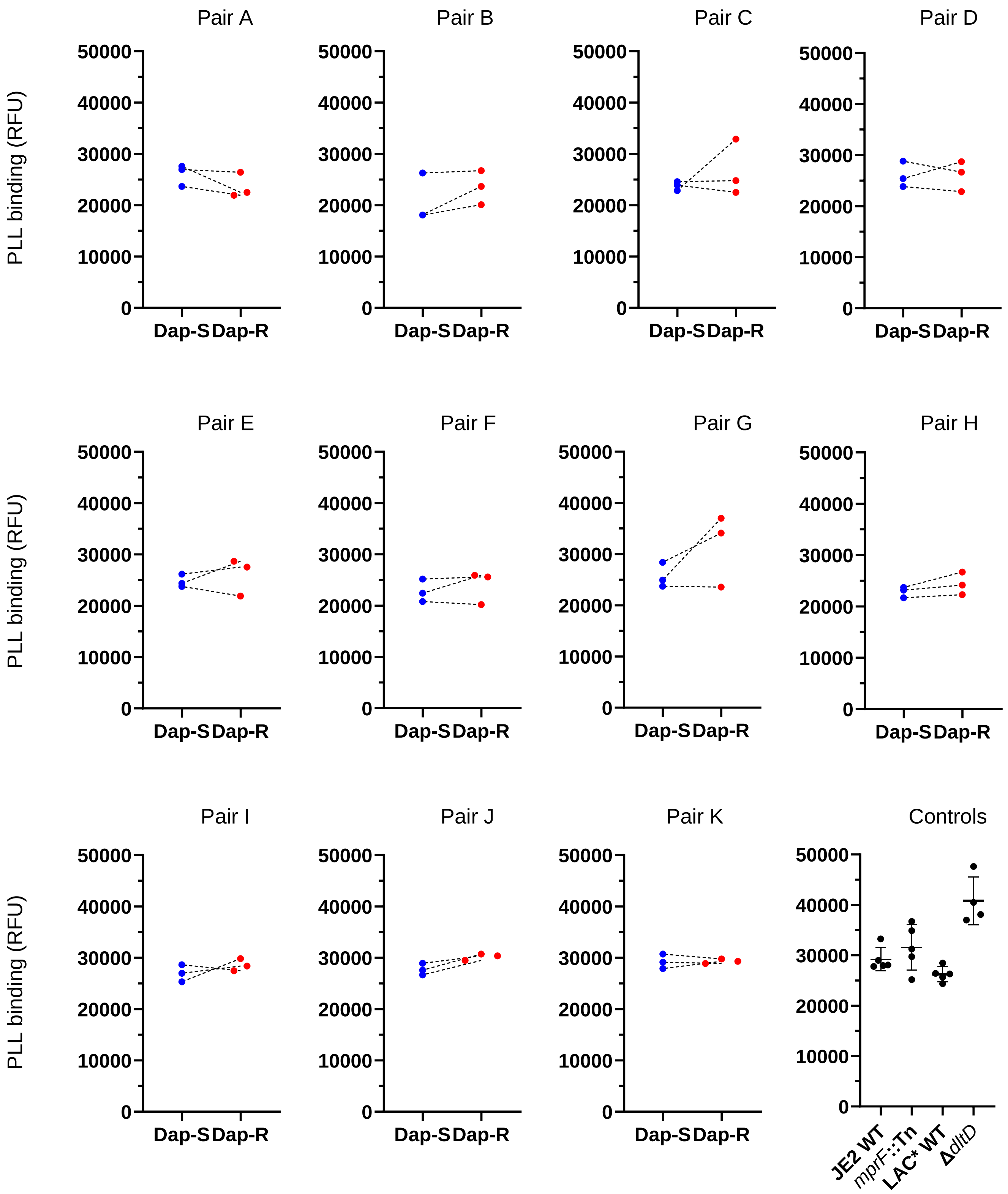

S2 Fig. Cell surface charge correlates with neither daptomycin resistance nor virulence in the fly. Cell surface charge of overnight TSB grown cells as determined by binding of highly positively charged fluorescein isothiocyanate-poly-L-lysine (FITC-PLL). Data from at least three independent experiments. Dashed lines link plots from same experiment. PBS, wild type USA 300 LAC*, USA 300 LAC JE2, USA 300 LAC JE2 mprF::Tn, USA 300 LAC* ΔdltD) served as controls. Data were analysed by a two-tailed paired Student's t-test. Difference in cell surface charge between paired strains is evidenced for none pair (p>0.05, all pairs) but pair J (p=0.0295). TSB: Trypic soy broth; RFU: relative fluorescence units; Dap-S, daptomycin susceptible; Dap-R, daptomycin resistant.

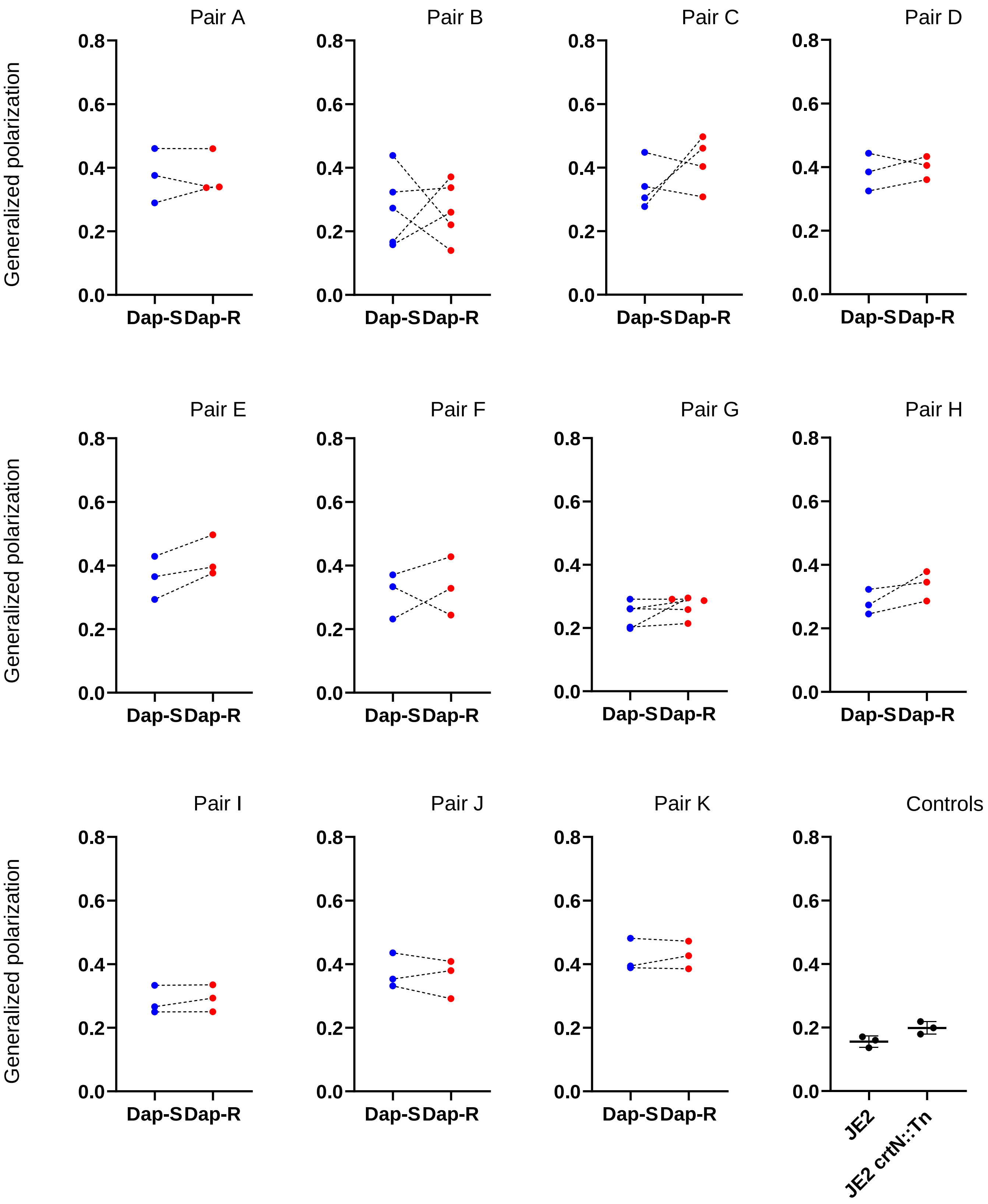

S3 Fig. Fluidity of cell membrane correlates with neither daptomycin resistance nor virulence in the fly. Membrane fluidity was determined using the fluorescent Laurdan dye to generate Generalised Polarisation. Data from at least three independent experiments. Dashed lines link plots from same experiment. Wild type USA 300 LAC JE2 and USA 300 LAC JE2 crtN::Tn were included in the series as control strains. Data were analysed by a two-tailed unpaired Student's t-test. Difference in membrane fluidity between paired strains is evidenced in none pair ($p>0.05$ for all pairs). RFU: relative fluorescence units; Dap-S, daptomycin susceptible; Dap-R, daptomycin resistant.

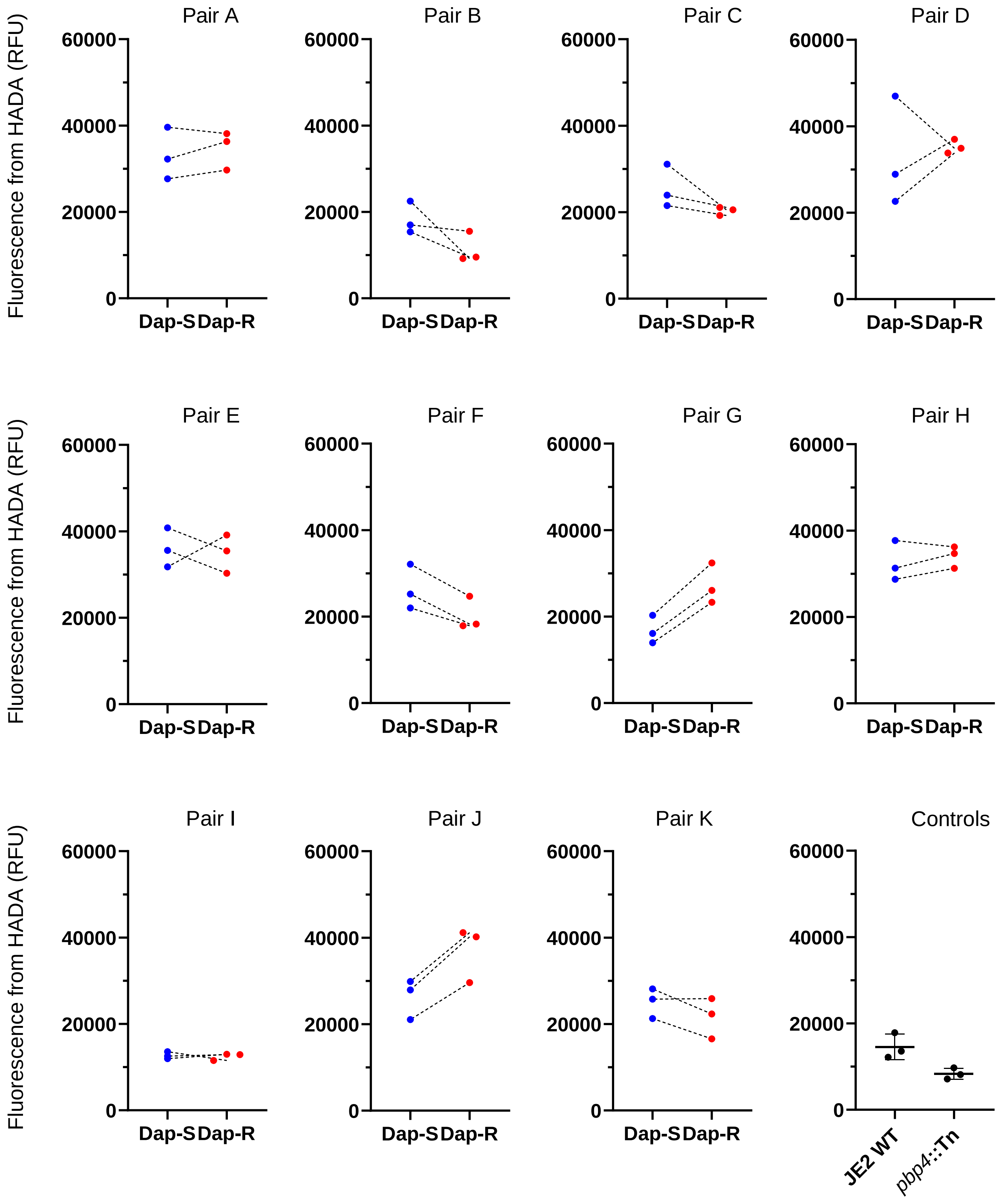

S4 Fig. Cell wall thickness correlate with neither daptomycin resistance nor virulence in the fly. Cell wall thickness was determined by the intake of a fluorescent peptidoglycan precursor (HADA) in cell wall of overnight grown cells in TSB with HADA. Data from at least three independent experiments are shown. Dashed lines link plots from same experiment. Wild type USA 300 LAC JE2 and USA 300 LAC JE2 *pbp4*::Tn were included in the series as control strains. Data were analysed by a two-tailed unpaired Student's t-test. No difference in cell wall thickness is evidenced between paired strains for pairs A-E, H, I, K (p>0.05) while a difference is observed for pair G (p=0.0330). TSB: Trypic soy broth; RFU: relative fluorescence units; Dap-S, daptomycin susceptible; Dap-R, Daptomycin resistant.

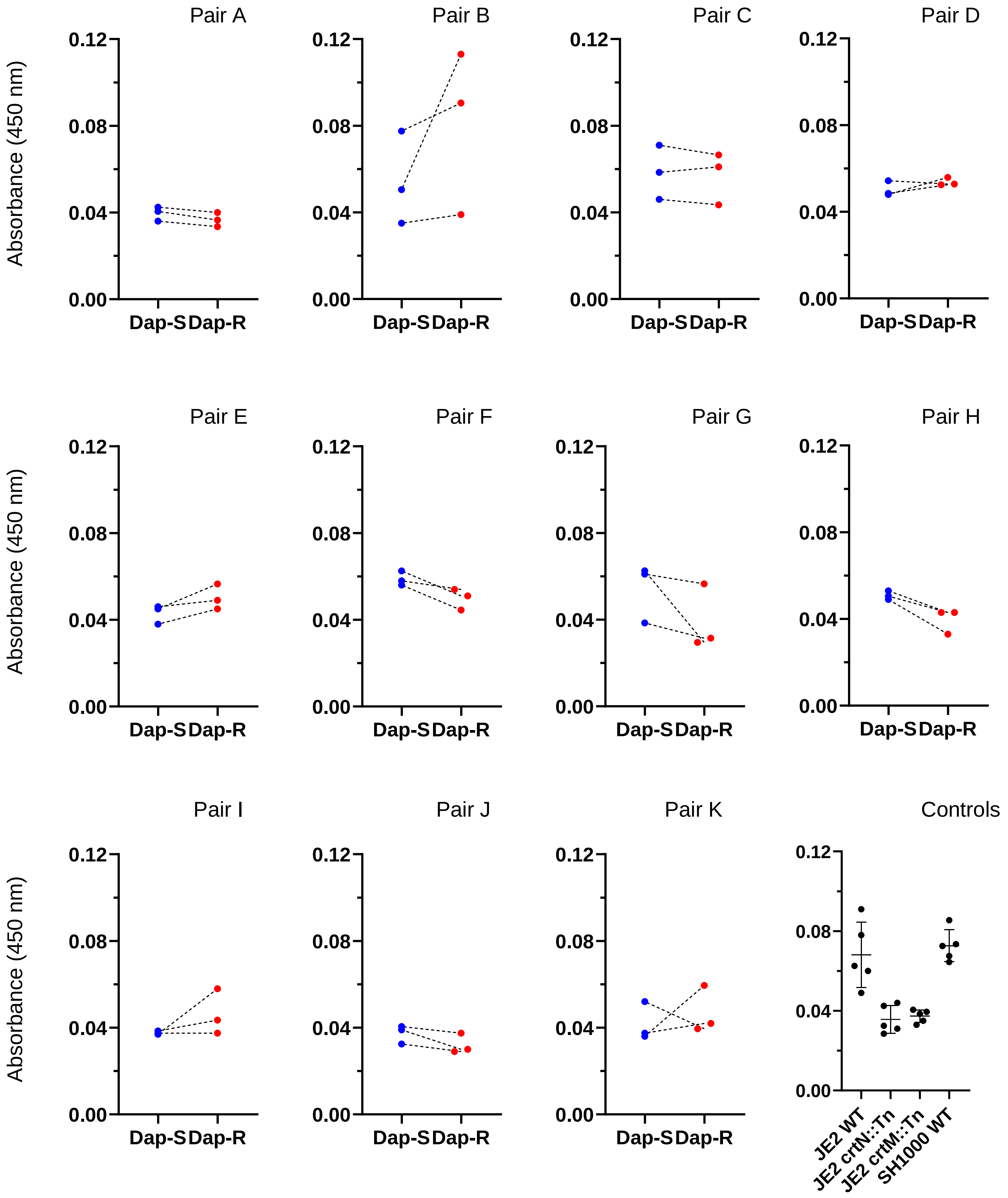

S5 Fig. Cell surface carotenoid content correlates with neither daptomycin resistance nor virulence in the fly. Data from three independent experiments. Dashed lines links data from same experiment. Wild type USA 300 LAC JE2, USA 300 LAC JE2 crtN::Tn, USA 300 LAC JE2 crtM::Tn, SH1000 were included in the series as control strains. Data were analysed by a two-tailed unpaired Student's t-test. Difference in cell surface carotenoid content between paired strains is evidenced for pair A (p=0.0267) and pair H (p=0.0341), for all other pairs p>0.05. RFU: relative fluorescence units; Dap-S, daptomycin susceptible; Dap-R, daptomycin resistant.

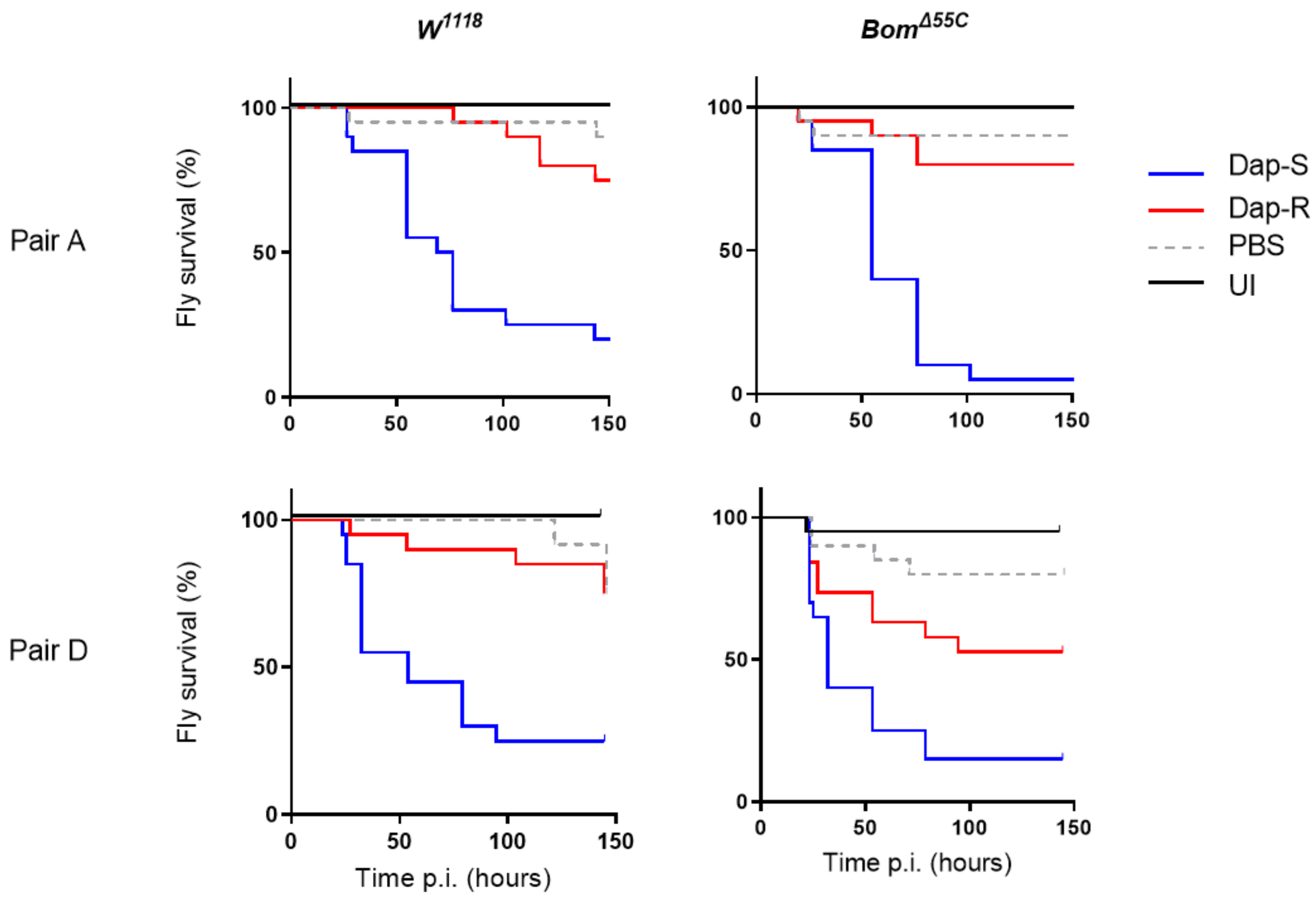

S6 Fig. Bomanins do not affect staphylococcal virulence depending on daptomycin resistance. Survival of flies infected with either Dap-S strain or Dap-R paired strain. Flies from two fly lineages were infected: wild type (*W*^{1118}) and bomanin- deficient (*Bom*$^{\Delta 55C}$, IM2) flies. Data presented for 2 pairs, pair A and pair D. Data from one of at least two independent experiments with similar results are presented. Data were analysed using the log-rang test: pair A, $p<0.0001$ (*W*^{1118}) and $p<0.0001$ (*Bom*$^{\Delta 55C}$); pair D, $p=0.0005$ (*W*^{1118}) and $p=0.0125$ (*Bom*$^{\Delta 55C}$). Dap-S, daptomycin susceptible; Dap-R, Daptomycin resistant.

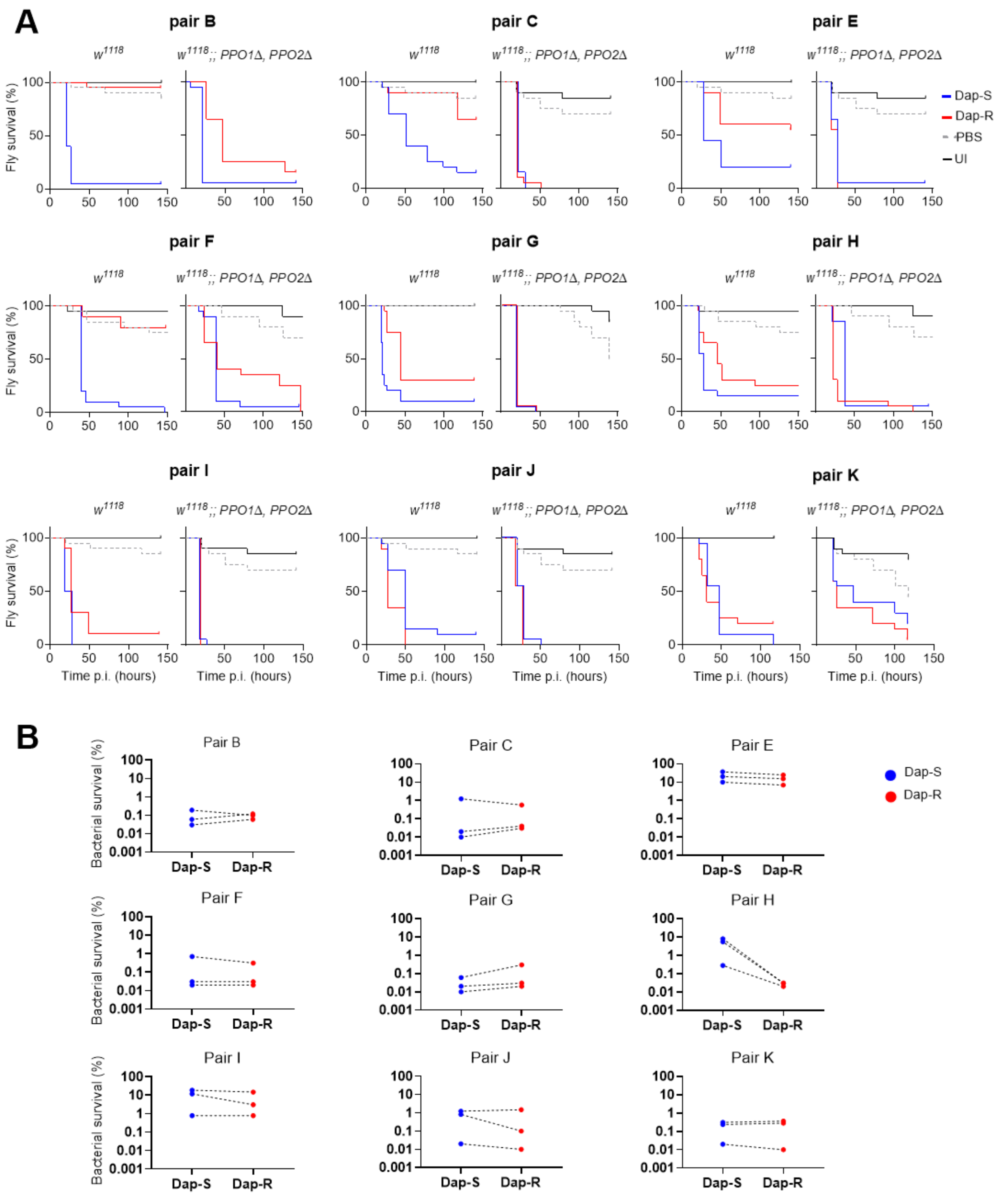

S7 Fig. Daptomycin resistant staphylococcal associated virulence is affected by prophenoloxidase but cells are not more susceptible to quinones. (A) Survival of wild type (W^{1118}) and W^{1118};; *PPO1Δ, PPO2Δ* mutants flies infected with either Dap-S strain or Dap-R paired strain. Lifespan analysis shows a consistent reduced difference in mortality of *PPO1Δ, PPO2Δ* double mutant flies between animals infected with Dap-S and Dap-R strains compared to wild type flies. A notable exception concerns pairs j and k that exhibit limited difference in virulence of the Dap-S and Dap-R paired strains in the wild type flies. Data from one of two independent experiments with similar results are presented. W^{1118}: log-rang test, Pairs B, C, F, and J, p≤0.0001, pair E, p=0.027, pair G, p=0.001, pair H, p=0.039, pair I: 0.15, pair K, p=0.22; PBS and UI, P>0.05. W^{1118}: Pairs B, C, F, and J, p≤0.0001, pair E, p=0.027, pair G, p=0.001, pair H, p=0.039, pair I: 0.15, pair K, p=0.22; PBS and UI, P>0.05; *PPO1Δ, PPO2Δ:* Pairs B, C, F, G, and I, p≤0.0001, pair E, p=0.0002, pair H, p=0.0007, pair J, p=0.0018, pair K: 0.0565, PBS and UI, P>0.05. **(B)** *In vitro* Survival of Dap-S and Dap-R paired strain of pairs other than pairs a and d exposed to ortho-benzoquinone (16 mg/l) for 1 hour, as determined by CFU counts. Survival does not correlate with bacterial virulence in the fly (two-tailed paired Student's t-test, p>0.05 for all pairs). Dap-S, daptomycin susceptible; Dap-R, Daptomycin resistant; CFU, colony forming unit.
