# Supplementary material for: Acquisition of daptomycin resistance in patients results in decreased virulence in Drosophila
Source: Infect Immun. 2025 May 23;93(6):e00594-24. doi: 10.1128/iai.00594-24 (PMC12150764; doi:10.1128/iai.00594-24)
Supplement: Supplemental tables — Tables S1 to S3. [file iai.00594-24-s0002.docx]

**S1 Table. Bacterial strains used in this study.**

| Strain | Description | Pair | Source |
| --- | --- | --- | --- |
| 244 | Recovered from blood culture, 2020 | a | This study |
| 225 | Recovered from blood culture, 2021 |  | This study |
| 1659 | Recovered from cerebrospinal fluid, 2017 | b | This study |
| 1658 | Recovered from cerebrospinal fluid, 2017 |  | This study |
| 287 | Recovered from blood culture, 2015 | c | This study |
| 288 | Recovered from blood culture, 2015 |  | This study |
| 1642 | Recovered from blood culture, 2017 | d | [1] |
| 1644 | Recovered from blood culture, 2017 |  | [1] |
| 611 | Recovered from heart valve, 2020 | e | This study |
| 408 | Recovered from blood culture, 2020 |  | This study |
| 655 | Recovered from blood culture, 2016 | f | This study |
| 654 | Recovered from blood culture, 2016 |  | This study |
| 1810 | Recovered from blood culture, 2017 | g | This study |
| 1811 | Recovered from blood culture, 2017 |  | This study |
| 260 | Recovered from joint fluid, 2014 | h | This study |
| 261 | Recovered from bile fluid, 2015 |  | This study |
| 2004 | Recovered from blood culture, 2018 | i | This study |
| 1963 | Recovered from blood culture, 2018 |  | This study |
| 1980 | n.d., 2014 | j | This study |
| 1981 | n.d., 2014 |  | This study |
| 1745 | Recovered from blood culture, 2020 | k | This study |
| 1688 | Recovered from blood culture, 2020 |  | This study |
| USA300 JE2 | LAC strain of the USA 300 CA-MRSA lineage, cured of plasmids | NA | [2] |
| USA300 JE2 mprF::Tn | USA300 LAC JE2 with a *bursa aurealis* transposon insertion in *mprF*, Ery^R^ | NA | [2] |
| *USA300 LAC* ΔdltD* | USA300 LAC* with the *dltD* gene deleted, Ery^R^ | NA | [3] |
| USA300 LAC* | LAC strain of the USA 300 CA-MRSA lineage, cured of LAC-p03 plasmid | NA | [4] |
| *USA300 LAC JE2 crtN::Tn* | USA300 LAC JE2 with a *bursa aurealis* transposon insertion in *crtN*, Ery^R^ | NA | [2] |
| *USA300 LAC JE2 crtM::Tn* | USA300 LAC JE2 with a *bursa aurealis* transposon insertion in *crtM*, Ery^R^ | NA | [2] |
| *USA300 LAC JE2 pbp4::Tn* | USA300 LAC JE2 with a *bursa aurealis* transposon insertion in pbp4, Ery^R^ | NA | [3] |
| SH1000 | Functional rsbU derivative of 8325-4 *rsbU+* | NA | [5] |

**References**

1. Dortet L, Anguel N, Fortineau N, Richard C, Nordmann P. In vivo acquired daptomycin resistance during treatment of methicillin-resistant Staphylococcus aureus endocarditis. International Journal of Infectious Diseases. 2013;17: e1076–e1077. doi:10.1016/j.ijid.2013.02.019

2. Fey PD, Endres JL, Yajjala VK, Widhelm TJ, Boissy RJ, Bose JL, et al. A Genetic Resource for Rapid and Comprehensive Phenotype Screening of Nonessential Staphylococcus aureus Genes. Bush K, editor. mBio. 2013;4: e00537-12. doi:10.1128/mBio.00537-12

3. Ledger EVK, Mesnage S, Edwards AM. Human serum triggers antibiotic tolerance in Staphylococcus aureus. Nat Commun. 2022;13: 2041. doi:10.1038/s41467-022-29717-3

4. Boles BR, Thoendel M, Roth AJ, Horswill AR. Identification of Genes Involved in Polysaccharide-Independent Staphylococcus aureus Biofilm Formation. Ratner AJ, editor. PLoS ONE. 2010;5: e10146. doi:10.1371/journal.pone.0010146

5. Horsburgh MJ, Aish JL, White IJ, Shaw L, Lithgow JK, Foster SJ. ␴B Modulates Virulence Determinant Expression and Stress Resistance: Characterization of a Functional rsbU Strain Derived from Staphylococcus aureus 8325-4. J BACTERIOL. 2002;184.

**S2 Table. Fly lines used in this study and their sources**

| **Line** | **Genotype** | **Description** | **Source** |
| --- | --- | --- | --- |
| W^1118^ | *W^1118^* | Isogenic wild type control | Bloomington *Drosophila* stock Center (BDSC) |
| Dif | *W^1118^ ; Dif^1^ cn bw* | Line carrying loss of function allele of *Dif* | D. Ferrandon, IBMC, Strasbourg |
| BomΔ55c | *W^1118^ ; Bom^Δ55c^* | Line lacking 10/12 Bomanin effector peptides | S. Wasserman, UCSD, San Diego |
| c564-Gal4 | *W^1118^ ;* c564-Gal4 | Fat body specific Gal4 driver line | BDSC |
| UAS-Toll^10B^ | *W^1118^ ;* UAS-*Toll^10B^/*FM7h | Line expressing constitutively active Toll under Gal4 control | BDSC |
| PPO1Δ, PPO2 Δ | *W^1118^;; PPO1Δ, PPO2 Δ* | Line lacking 2/3 prophenoloxidase activities | B. Lemaitre, EFPL, Lausanne |

UCSD: University of San diego ; IBMC: Institut de biologie moléculaire et cellulaire ; EFPL: Ecole polytechnique fédérale de Lausanne

**S3 Table. List of RT-qPCR primers used for expression analysis of *Drosophila* antimicrobial effectors in this study**

| **Gene** | **Identifier** | **Forward** | **Reverse** |
| --- | --- | --- | --- |
| Rpl4 | CG5502 | TCCACCTTGAAGAAGGGCTA | TTGCGGATCTCCTCAGACTT |
| BomS2 (*IM2*) | CG18106 | ACCGTCTTTGTGTTCGGTCT | GATTACCACATTTCCTGGATCG |
| *Drs* | CG10810 | GTACTTGTTCGCCCTCTTCG | CTTGCACACACGACGACAG |
| *AttA* | CG10146 | CACAATGTGGTGGGTCAGG | GGCACCATGACCAGCATT |
| *Mtk* | CG8175 | TCTTGGAGCGATTTTTCTGG | TCTGCCAGCACTGATGTAGC |
